# Supplementary material for: The Wnt/β-catenin/TCF/Sp5/Zic4 Gene Network That Regulates Head Organizer Activity in Hydra Is Differentially Regulated in Epidermis and Gastrodermis
Source: Biomedicines. 2024 Jun 8;12(6):1274. doi: 10.3390/biomedicines12061274 (PMC11201823; doi:10.3390/biomedicines12061274)
Supplement: Supplementary file 1 [file biomedicines-12-01274-s001.zip › biomedicines-3001436-supplementary.pdf]

The *Wnt/β-catenin/TCF/Sp5/Zic4* gene network that regulates head organizer activity in *Hydra* is differentially regulated in epidermis and gastrodermis

Laura Iglesias Ollé [1], Chrystelle Perruchoud [1], Paul Gerald Layague Sanchez [1], Matthias Christian Vogg [1] and Brigitte Galliot [1,*]

SUPPLEMENTARY TABLES

Table S1: Accession numbers (AN) of the *Hydra* genes used in this study p2

Table S2: List of primer, siRNA and ds-oligonucleotide sequences p3

Table S3: List of reporter or expression constructs used in this work p4

SUPPLEMENTARY FIGURES

Figure S1: *Sp5* (t29291aep) single-cell expression as deduced from *Hydra* single-cell transcriptomes; p5

Figure S2: Map and sequences of the *HyActin-1388*:mCherry_*HySp5-3169*:GFP construct (10'533 bp); p6

Figure S3: Epidermal and gastrodermal GFP fluorescence in intact *HySp5-3169*:GFP transgenic animals; p8

Figure S4: Epidermal and gastrodermal profiles of GFP and mCherry fluorescence in intact *HySp5-3169*:GFP transgenic animals; p9

Figure S5: Layer-specific *GFP* expression in regenerating *HySp5-3169*:GFP transgenic animals; p10

Figure S6: GFP and mCherry fluorescence in apical-regenerating (AR) or basal-regenerating (BR) halves of epidermal and gastrodermal *HySp5*-3169:GFP transgenic animals; p11

Figure S7: Impact of alsterpaullone (ALP) treatment on *Sp5* and *Wnt3* expression in non-transgenic *Hv_AEP2* animals; p12

Figure S8: Impact of ALP treatment on *Sp5, GFP* and *Wnt3* expression patterns in epidermal and gastrodermal *HySp5-3169*:GFP transgenic animals; p14

Figure S9: Impact of ALP treatment on *Sp5, GFP* and *Wnt3* expression patterns in epidermal and gastrodermal *HyWnt3-2149*:GFP transgenic animals; p16

Figure S10: Impact of *β-catenin*(RNAi) on *β-catenin*, *Sp5* and *GFP* transcript levels in epidermal and gastrodermal *HySp5-3169*:GFP transgenic animals; p17

Figure S11: Impact of *β-catenin*(RNAi) on formation of bud-like structures expressing *Sp5* in *Hv-Basel* animals p18

Figure S12: Impact of *β-catenin*(RNAi) on GFP and mCherry fluorescence in epidermal and gastrodermal *HySp5-3169*:GFP transgenic animals after one, two or three siRNA exposures; p19

Figure S13: Impact of *β-catenin*(RNAi) on GFP and mCherry immuno-detected patterns in epidermal and gastrodermal *HySp5-3169*:GFP transgenic animals six days after the 2nd siRNA exposure; p20

Figure S14: Impact of *Sp5*(RNAi) on *Sp5, GFP* and *Wnt3* transcript levels in epidermal and gastrodermal *HySp5-3169*:GFP transgenic animals; p21

Figure S15: Impact of *Sp5*(RNAi) on *GFP* expression patterns in epidermal and gastrodermal *HySp5-3169*:GFP transgenic animals; p23

Figure S16: Impact of *Sp5 (RNAi)* on GFP and mCherry fluorescence in epidermal or gastrodermal *HySp5-3169*:GFP transgenic animals at early time-points after one or two siRNA exposures; p25

Figure S17: Impact of *Sp5(RNAi)* on GFP and mCherry fluorescence in epidermal and gastrodermal *HySp5-3169*:GFP animals two days after the 2nd siRNA exposure; p26

Figure S18: ChIP-qPCR analysis of the Sp5-binding sites in the *HySp5* promoter using anti *HySp5* antibodies; p27

Figure S19: Mapping the putative *Sp5* Transcriptional Start Sites (TSS); p28

Figure S20: Putative *Zic4*-binding sites in the *Hydra Wnt3* and *Zic4* genomic sequences p29

SUPPLEMENTARY TABLES

Table S1: Accession numbers (AN) of the *Hydra* genes used in this study

Gene name	Hydratlas.unige *(Hv_Jussy, Hv_AEP1, Hol_CS,_CR)*	UNIPROT / NCBI *(H. vulgaris, Hm-105)*	single cell portal *(Hv_AEP2)*	Genomic DNA *(Hm-105)*
β-actin	seq16839_loc07321 (*Hv_Jussy)*<br>c25578_g1_i02 (*Hv_AEP1*)<br>S040281c2g2_i01 (*Hol_CS*)<br>R040833c2g5_i01 (*Hol_CR*)	P17126_HYDVU<br>XP_002154696.1	t11116aep	Sc4wPfr_135
β-catenin	seq73524_loc24304 (*Hv_Jussy*)<br>c12650_g1_i01 (*Hv_AEP1*)<br>S042301c1g1_i01 (*Hol_CS*)<br>R029792c0g1_i01 (*Hol_CR*)	T2M8K9_HYDVU<br>XP_012562750.1	t13357aep	Sc4wPfr_177
Sp5	seq62049_loc21222 (*Hv_Jussy*)<br>c16537_g1 (*Hv_AEP1*)<br>S034511c0g1_i01 (*Hol_CS*)<br>R029443c0g1_i01 (*Hol_CR*)	T2MC68_HYDVU<br>XM_004206770.3	t29291aep	Sc4wPfr_224.1
TBP	seq65236_loc22074 (*Hv_Jussy*)<br>c16197_g1_i01 (*Hv_AEP1*)<br>S023957c0g1_i01 (*Hol_CS*)<br>R001357c0g1_i01 (*Hol_CR*)	T2MG73_HYDVU<br>XP_002160884.2	t38685aep	Sc4wPfr_29
TCF	seq62874_loc21437(*Hv_Jussy*)<br>c21328_g1_i01(*Hv_AEP1*)<br>S036398c0g1_i05 (*Hol_CS)*<br>R035844c0g2_i01 (*Hol_CR*)	Q9GTK1_HYDVU<br>NP_001296662.1	t11826aep	Sc4wPfr_319
Wnt3	*seq49770*_loc17775<br>(*Hv_Jussy*)<br>c3776_g1_i01 (*Hv_AEP1*)<br>S029978c0g1_i01 (*Hol_CS*)<br>R033453c0g1_i01 (*Hol_CR*)	Q9GTJ9_HYDVU<br>NP_001274292.1	t14194aep	Sc4wPfr_399
Wnt5a	*seq29395_loc11578*<br>(*Hv_Jussy*)<br>*c19333_g1_i01* (*Hv_AEP1*)<br>*S037476c0g1_i02* (*Hol_CS*)<br>*R009822c0g1_i01* (*Hol_CR*)	A0A8B6XQ36_HYDVU<br>NP_001296688.1	t21554aep	Sc4wPfr_287.2
Wnt8	*seq25361_loc10235*<br>(Hv_Jussy)<br>*c12431_g1_i01* (Hv_AEP1)<br>*S039485c1g1_i01* (Hol_CS)<br>*R036877c0g1_i01* (Hol_CR)	A0A8B7DSN5_HYDVU<br>XP_047127276.1	t23521aep	Sc4wPfr_440
Zic4	seq19466_loc08275 (*Hv_Jussy*)<br>c11132_g1_i01 (*Hv_AEP1*)<br>S030492c0g1_i01 (*Hol_CS*)<br>R028263c0g1_i01 (*Hol_CR*)	T2M6U0_HYDVU<br>XP_002153782.2	t20709aep	Sc4wPfr_237.2

Table S2: List of primer, siRNA and ds-oligonucleotide sequences

**Cloning primers**	HySp5 promoter Forward	CCGGATATCCTAGTTCTAATTTAGCTCTATTACGTTCGC
	HySp5 promoter Reverse	AACCCCTTATCAAAGAAGCCACCGGTCTAG
**siRNAs**	HySp5 siRNA-1	UUA ACG AGC ACC ACA UAA A
	HySp5 siRNA-2	CUA CAA CAU CCC ACA UAU A
	HySp5 siRNA-3	GCA GCA CGU AUG UCA UAU U
	β-catenin siRNA-1	UCA ACC UAA CAG ACA ACA A
	β-catenin siRNA-2	UGA GGA GCU AUA CUU AUG A
	β-catenin siRNA-3	ACG ACU CUC UGU UGA AUU U
	Scramble siRNA	AGG UAG UGU AAU CGC CUU G
**qPCR primers**	HySp5 Forward	CCAGGGTGCGGAAAGGTT
	HySp5 Reverse	CCAGCATGCCATCTTAAATGAG
	HyWnt3 Forward	GAGTTGACGGTTGCGAACTT
	HyWnt3 Reverse	ACATGAAACCTTGCAACACCA
	β-catenin Forward	TACGCAATGTTGTTGGTGCT
	β-catenin Reverse	GCTTCAATTCGATGGCCTAA
	GFP Forward	TGGAAGCGTTCAACTAGCAG
	GFP Reverse	AAAGGGCAGATTGTGTGGAC
	TBP Forward	AAGCGATTTGCAGCAGTTAT
	TBP Reverse	GCTCTTCACTTTTTGCTCCA
**qPCR primers for ChIP**	Sp5prom_F_1	TAAGCTGTCTCCATTTCAACCA
	Sp5prom_R_1	AATATTTGTTAAGTGTTTTTCGTTGG
	Sp5prom_F_2	AATTGCGGTAAAGATCAGTAAGAA
	Sp5prom_R_2	TGGTTGAAATGGAGACAGCTT
	Sp5prom_F_3	AAGTATCAAGTTTAAAAATTCCTTCG
	Sp5prom_R_3	ATTTAGAATTCTTACTGATCTTTACCG
	Sp5prom_F_4	TATCTTTTCCGCCTTACGTATTC
	Sp5prom_R_4	ACTGAGAAATGGCGCGTTG
	Sp5prom_F_5	CAGAGAAAATATGATCGCAACG
	Sp5prom_R_5	GAAACCGCCATCTTATCTTAAA
	Sp5prom_F_6	AACCAAATATTTAAAATGATAAACTGG
	Sp5prom_R_6	CAAAGGCGGAGTAATTAGGTG
	Sp5prom_F_7	TGATTTGAAGTCAAAAACAAAATAACA
	Sp5prom_R_7	TGGTTAAAAGATATAAAACGCTATTTG
	Sp5prom_F_8	TGGTAAAGTTCGTAAAACCAATGA
	Sp5prom_R_8	AAACATTCCGACAATCCACAG
	Sp5prom_F_9	AAGTAGCGACAGCGCCAGT
	Sp5prom_R_9	ATATCCTAGCCAAAACAAAACAA
	Sp5prom_F_10	GGTCAGCGAGTTTGGATCAT
	Sp5prom_R_10	AGCCTCAGGACTTCCCATTT
	Sp5prom_F_11	CGAGCGTTGCTTTGACTTTA
	Sp5prom_R_11	CAATTACGGATCACCGAAGG
	Sp5prom_F_12	CTCAGTGCATCCGTTCGTT
	Sp5prom_R_12	TCTTGCTTGCTTACGGATGA
	Sp5prom_F_13	TGAAATATTAAAAAGACGGAAGGAA
	Sp5prom_R_13	TGCAGTGAAAAGCAACAAACA
	Sp5prom_F_14	CGTTCGCAAAGTTGACAAAGT
	Sp5prom_R_14	CAATTTTTATAAGCGTGATAAAGCAA
	Sp5prom_F_15	TCAATTTCAACAAAATAAGTGTCAA
	Sp5prom_R_15	TGAAGTTTCAATCCCTTTTAAACAA
**double-stranded oligos for EMSA**	ds-Sp5-oligo PPA wt	TATCTTTT**CCGCCT**TACGTATTCTGTTTATCA**CCGCCT**CTTAGACCATCCCATTTGTACGTAAACAGAG
	ds-Sp5-oligo PPA mut	TATCTTTT**CTTCCT**TACGTATTCTGTTTATCA**CTTCCT**CTTAGACCATCCCATTTGTACGTAAACAGAG
	ds-Sp5-oligo PPB wt	CAGAGAAAATATGATCGCAAC**GCGCCA**TTTCTCAGTCAG**AGGCGT**GACATTAACCCCTTATCAAAGAAGCCGA
	ds-Sp5-oligo PPB mut	CAGAGAAAATATGATCGCAAC**GTTCCA**TTTCTCAGTCAG**ATTCGT**GACATTAACCCCTTATCAAAGAAGCCGA

Table S3: List of reporter or expression constructs used in this work

PLASMID NAME	PLASMID DESCRIPTION	REFERENCE
**CONSTRUCTS TO BE EXPRESSED *EX-VIVO***		
***HySp5-2992*:Luciferase** in pGL3	Construct where 2992 bp *Hydra Sp5* upstream sequences directing luciferase expression in human HEK293T cells	[33]
***HySp5-2828*:Luciferase** in pGL3	Construct derived from the *HySp5-2992*:Luciferase plasmid after deleting 164 bp of the proximal *Hy Sp5* promoter sequences	This work (Fig. 6, Fig. 7)
HySp5-2992-MBS1*:Luc**<br>HySp5-2992-MBS2*:Luc**<br>***HySp5-2992-MBS3*:Luc**<br>***HySp5-2992-MBS4*:Luc**<br>***HySp5-2992-MBS5*:Luc**	Constructs derived from the *HySp5-2992*:Luciferase construct after mutating in each one out of the 5 Sp5-binding sites located in the proximal promoter: BS1 at position -129, BS2 at position -105, BS3 at position -52, BS4 at position -34, BS5 at position +18	This work (Fig. 6, Fig. 7)
CMV*:HySp5-420** in pCS2+<br>CMV*:HySp5-337-δDBD**	Expression constructs designed to express either full length Sp5 (HySp5-420) or Sp5-337 lacking its DNA-binding domain (ΔDBD)	[33]
**pCS2+-HySp5-420**	bacterial vector to express the *Hydra* Sp5 protein, here full length	[33]
***HyWnt3-2149*:Luciferase** in pGL3	Construct where 2149 bp *Hydra Wnt3* upstream sequences directing luciferase expression in human HEK293T cells	[33]
***CMV*:hu_β-CateninΔ45** in pFLAG	Construct designed to express a truncated version of β-catenin protein, constitutively active in human cells	[78]
**TOPFLASH**<br><br>**FOPFLASH**	TOPFLASH: Reporter construct where 6 arrowed consensus TCF binding sites upstream of the minimal TK promoter direct Luciferase expression; FOPFLASH: Reporter construct where 6 arrowed mutated TCF binding sites upstream of the minimal TK promoter direct Luciferase expression	[79]
**pCAG-FLAG-TCF-1**	Construct designed to express a FLAG-tagged version of the human TCF1 transcription factor in human cells	[80]
***HyZic4-3505*:Luciferase** in pGL3	Reporter construct where 3505 bp of the *HyZic4* upstream sequences drive luciferase expression in human HEK293T cells	[34]
CMV*:HyZic4-431** in pCS2+<br>CMV*:HyZic4-ΔDBD**	Expression constructs designed to express Zic4 either full length (HyZic4-431) or lacking its DNA-binding domain (HyZic4-ΔDBD)	[34]
**CONSTRUCTS TO BE EXPRESSED IN HYDRA**		
***HyAct-1388*:eGFP (**hoTG**)**	Transformation reporter construct where 1289 bp upstream and 99 bp 5'UTR sequences of *Hydra Actin* direct ubiquitous GFP expression (full sequence available @ NCBI: DQ369740)	[39]
**hoTG-*HyWnt3FL*:eGFP-*HyAct*:dsRED**	Tandem reporter construct where 2149 bp upstream sequences of *HyWnt3* direct eGFP expression and 1'289 bp upstream sequences of *HyActin* direct ubiquitous RFP expression	[27]
***HyAct-1388*:mCherry::*HySp5-3169*:eGFP** in PBSSA	Tandem reporter construct where 1289 bp upstream and 99 bp 5'UTR sequences of *Hydra Actin* direct mCherry expression and 3'169 bp upstream sequences of *Hydra Sp5* direct eGFP expression	This work (Fig. S2)
**CONSTRUCTS TO DETECT GENE EXPRESSION IN HYDRA**		
GFP-663*_*pGEM-T-Easy	Plasmid to produce a 663 nt-long *GFP* riboprobe	This work
HySp5-502*_*pGEM-T-Easy	Plasmid to produce a 502 nt-long *Sp5* riboprobe	[33]
HyWnt3-1092*_*pGEM-T-Easy	Plasmid to produce a 1092 nt-long *Wnt3* riboprobe	[33]

SUPPLEMENTARY FIGURES

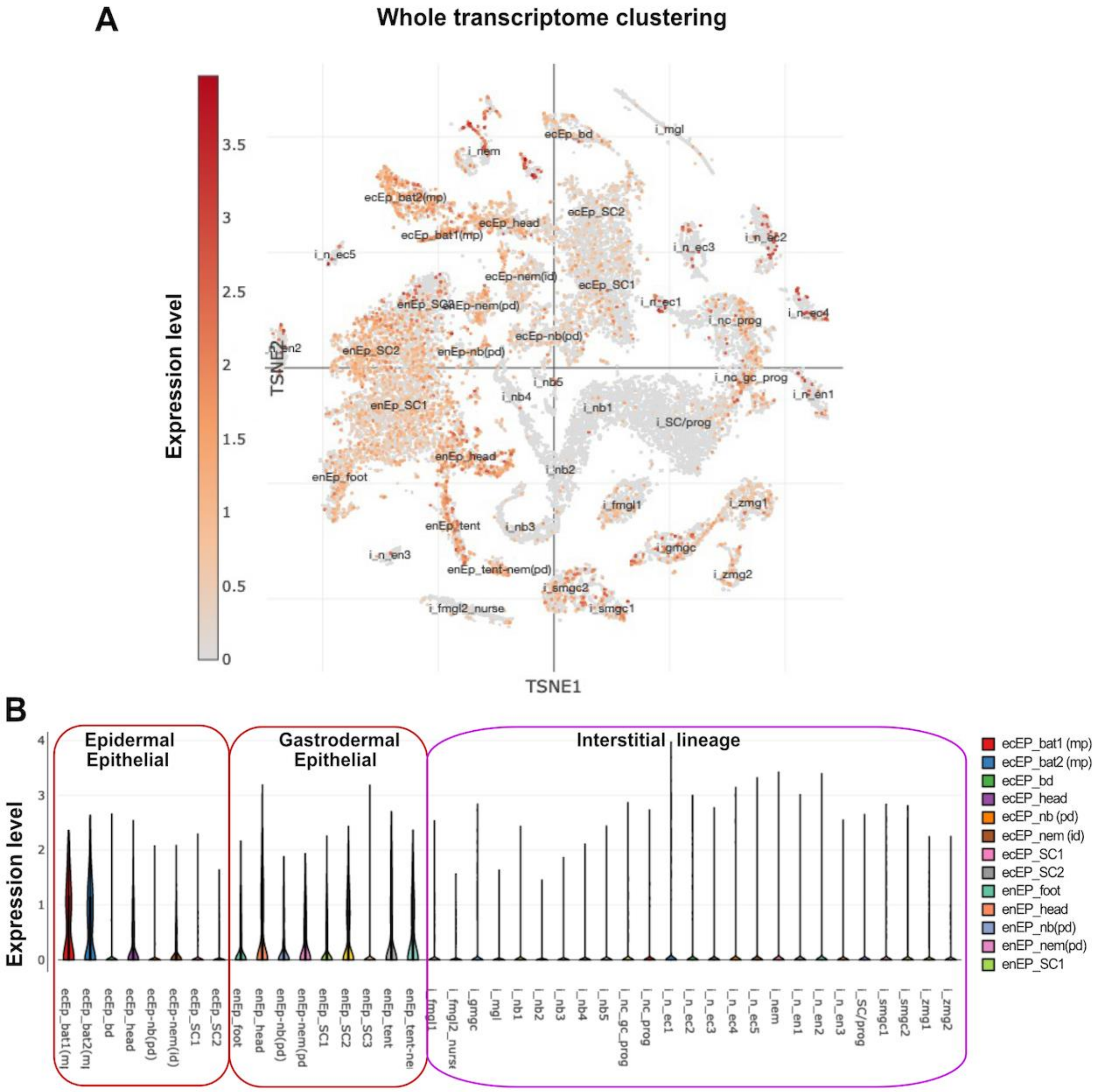

Figure S1. ***Sp5*** **(t29291aep) single-cell expression as deduced from** ***Hydra*** **single-cell transcriptomes**

Hydra single-cell transcriptomes were produced by Siebert et al (2019) and made available on Single Cell Portal. **(A)** tSNE plot of SNN clustered single cell data mapped to the LRv2 transcriptome reference. **(B)** Distribution of *Sp5* transcripts per cell type. The *Hydra Sp5* sequence from the *Hv_AEP* strain (c16537_g1 available on HydrAtlas) was used for a blast search on the Juliano aepLRv2 reference transcriptome. The protein sequences deduced from c16537_g1/Sp5 and t29291aep/Sp4 cDNAs are 100% identical. Note the predominant expression in the epithelial cells (Ep) from the epidermis (also called ectoderm, ecEP) or the gastrodermis (also called endoderm, enEp). Epithelial sub-populations were identified as stem cells (epEP_SC1, epEP_SC2, enEp_SC1, enEp_SC2, enEp_SC3), suspected phagocytosis doublet (ecEp-nb(pd), enEp-nb(pd), enEp-nem(pd)), epidermal cells that contain integration doublet (ecEp-nem(id)), tentacle-specific in the gastrodermis (enEp_tent, enEp_tent-nem), tentacle-specific epidermal battery cells that are multiplet (ecEP_bat1(mp), ecEP_bat2(mp)), apical-specific (ecEp_head, enEp_head), basal-specific (ecEp_bd, enEp_foot). **Abbreviations: bat:** battery cell, **bat(mp):** multiplet battery cells, **bd:** basal disc, **ec:** ectoderm (=epidermis), **en:** endoderm (=gastrodermis), **ep:** epithelial, **fmgl:** female germline, **gc:** gland cell, **gmgc:** granular mucous gland cell, **i:** cell from the interstitial lineage; **id:** integration doublet, **mgl:** male germline, **mp:** multiplet, **nb:** nematoblast, **n**: neuronal cell, **nem:** nematocyte, **pd:** suspected phagocytosis doublet, **prog:** progenitors, **SC:** Stem Cell, **smgc:** spumous mucous gland cell, **tent:** tentacle, **zmg:** zymogen gland cell.

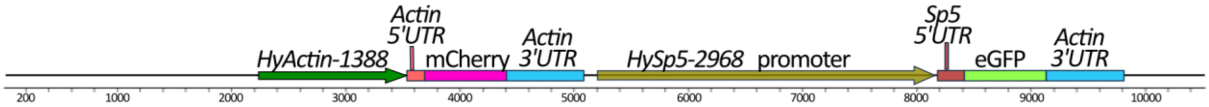

pBSSA-AR plasmidic sequence (2'244 bp)

1 gtggcacttttcggggaaatgtgcgcggaacccctatttgtttatttttctaaatacattcaaatatgtatccgctcatgagacaataaccctgataaat
101 gcttcaataatattgaaaaaggaagagtatgagtattcaacatttccgtgtcgcccttattcccttttttgcggcattttgccttcctgtttttgctcac
201 ccagaaacgctggtgaaagtaaaagatgctgaagatcagttgggtgcacgagtgggttacatcgaactggatctcaacagcggtaagatccttgagagtt
301 ttcgccccgaagaacgttttccaatgatgagcacttttaaagttctgctatgtggcgcggtattatcccgtattgacgccgggcaagagcaactcggtcg
401 ccgcatacactattctcagaatgacttggttgagtactcaccagtcacagaaaagcatcttacggatggcatgacagtaagagaattatgcagtgctgcc
501 ataaccatgagtgataacactgcggccaacttacttctgacaacgatcggaggaccgaaggagctaaccgcttttttgcacaacatgggggatcatgtaa
601 ctcgccttgatcgttgggaaccggagctgaatgaagccataccaaacgacgagcgtgacaccacgatgcctgtagcaatggcaacaacgttgcgcaaact
701 attaactggcgaactacttactctagcttcccggcaacaattaatagactggatggaggcggataaagttgcaggaccacttctgcgctcggcccttccg
801 gctggctggtttattgctgataaatctggagccggtgagcgtgggtctcgcggtatcattgcagcactggggccagatggtaagccctcccgtatcgtag
901 ttatctacacgacggggagtcaggcaactatggatgaacgaaatagacagatcgctgagataggtgcctcactgattaagcattggtaactgtcagacca
1001 agtttactcatatatactttagattgatttaaaacttcatttttaatttaaaaggatctaggtgaagatcctttttgataatctcatgaccaaaatccct
1101 taacgtgagttttcgttccactgagcgtcagaccccgtagaaaagatcaaaggatcttcttgagatcctttttttctgcgcgtaatctgctgcttgcaaa
1201 caaaaaaaccaccgctaccagcggtggtttgtttgccggatcaagagctaccaactctttttccgaaggtaactggcttcagcagagcgcagataccaaa
1301 tactgtccttctagtgtagccgtagttaggccaccacttcaagaactctgtagcaccgcctacatacctcgctctgctaatcctgttaccagtggctgct
1401 gccagtggcgataagtcgtgtcttaccgggttggactcaagacgatagttaccggataaggcgcagcggtcgggctgaacggggggttcgtgcacacagc
1501 ccagcttggagcgaacgacctacaccgaactgagatacctacagcgtgagctatgagaaagcgccacgcttcccgaagggagaaaggcggacaggtatcc
1601 ggtaagcggcagggtcggaacaggagagcgcacgagggagcttccagggggaaacgcctggtatctttatagtcctgtcgGgtttcgccacctctgactt
1701 gagcgtcgatttttgtgatgctcgtcaggggggcggagcctatggaaaaacgccagcaacgcggcctttttacggttcctggccttttgctggccttttg
1801 ctcacatgttctttcctgcgttatcccctgattctgtggataaccgtattaccgcctttgagtgagctgataccgctcgccgcagccgaacgaccgagcg
1901 cagcgagtcagtgagcgaggaagcggaagagcgcccaatacgcaaaccgcctctccccgcgcgttggccgattcattaatgcagctggcacgacaggttt
2001 cccgactggaaagcgggcagtgagcgcaacgcaattaatgtgagttagctcactcattaggcaccccaggctttacactttatgcttccggctcgtatgt
2101 tgtgtggaattgtgagcggataacaatttcacacaggaaacagctatgaccatgattacgccaagcgcgcaattaaccctcactaaagggaacaaaagct
2201 ggtaccgggccccccctcgaggtcgactctagaggatccccccat 2244

Actin promoter sequences (1'289 bp) + Actin 5'UTR (99 bp) + <u>Actin coding sequence</u> (27 bp) + MCS (27 bp)

-1289 cgatctgactaacctaaccagtgcaaaaaaatttaaaagatttgcattgtgaaagttagaatattataaaaaatctaaaacgagtattactcgagtaaat
-1189 gttatacgatctatagattaaatatattaaaaatgtatagcgaatgttaaactaaatatataatataaacttgaaaacttactaaattgcaaaaactcaa
-1089 aaccgactgtatcattttacaggaaaccgttattcaagatacttaagttgtttactacattattataacatcttgcaattagcaagacaatcgttattt
-989 taacatcacggtatcgaaaggattttgagaaattttattgaaacattttaaacaaaaaatatcatatttagatgcattttaagccgagatgcaggattct
-889 gaatgaaaaagaaaaaaagaagtctcggtagagtaaaagtgatcggtttgcaactgtaaaatttattgaagtaccaataatttatttaaaataaaactg
-789 aaatataaagttaaagttgctgttctataagtttaccgaattttaaaaccattgtaacgctagagtaatatttgagtctactaagttagtccccgcactt
-689 tttaatcaagcaataaatacccaaactttgcttattcaaatcaataaaccaatatatctcttaaaataaagtaaaaacttctgaaattctataaaaaaaa
-589 atttaatttcgaaatatcaaatgtaacttcaacaccgcactattttcttttaaacaactgatatagtaattacttctcaaaaacgttatctcaaggtttg
-489 tgatgtacttaaaaccactcctattttgttacgcgtttaaaaaagcaaacataagttggtttctattgatgaatgagaacatatttcatttaaagttaaa
-389 atcctaccagtggtttcactgtacgtaaacaccgtcaaaaaaacaggaacgtttttaaagattaataattgaagtaaaaaaaatttaataccggggtta
-289 aaaaaatcttttaaaataattataaatatatatattaaaatttataaattttaaacacatttaaaatatatattaagtataataaaagtaatattataa
-189 aaaaaaatttaattttataattattttttattaaatttataaataataggtaaaacttacatatccgttttattttttcttaataaaataacgcgtgcaaa

TSS +1

-89 ttttgtccatataaagaccttttcgaacaataactttttgcttagccgtttttttcttatatggtcaaaaaagcgctcaagcgatt**CA**ccataaaaa

99

12 gcgcaattagttcagcgttcgttattcagaagcttcagctttgcttgatactcagctcttctctttttaaacaaaacacttaatcaaa<u>**ATG**GCCGATGAT</u>

M A D D

<u>GAAGTTGCCGCCCTC</u> GCTGCAGCCCCGGTAGAAAAAGTCGAC

E V A A L

mCherry coding sequence (711 bp) + MCS (14 bp)

1 ATGGTTAGTAAAGGAGAAGAAGATAATATGGCTATTATTAAAGAATTTATGAGATTTAAAGTTCATATGGAAGGATCTGTTAATGGTCATGAATTTGAAA
101 TTGAAGGTGAAGGAGAAGGTAGACCATATGAAGGTACTCAAACAGCTAAACTTAAAGTTACTAAAGGTGGACCATTACCTTTTGCATGGGATATTCTTTC
201 ACCACAATTTATGTATGGAAGTAAAGCTTATGTTAAACATCCAGCAGATATTCCTGATTATCTTAAACTTTCATTTCCTGAAGGTTTTAAATGGGAAAGA
301 GTTATGAATTTTGAAGATGGTGGAGTTGTTACTGTTACACAAGATTCTTCATTACAAGATGGAGAATTTATTTATAAAGTTAAACTTAGAGGAACTAATT
401 TTCCAAGTGATGGTCCTGTTATGCAAAAGAAAACTATGGGTTGGGAAGCTAGTTCTGAAAGAATGTATCCTGAAGATGGAGCACTTAAAGGTGAAATTAA
501 ACAAAGACTTAAACTTAAAGATGGTGGACATTATGATGCTGAAGTTAAAACTACATATAAAGCTAAAAAACCAGTTCAATTACCTGGAGCTTATAATGTT
601 AATATTAAACTTGATATTACTTCTCATAATGAAGATTATACAATTGTTGAACAATATGAAAGAGCAGAAGGTAGACATTCAACAGGTGGAATGGATGAAT
701 TATATAAATAA CATTCGTAGAATTC

Actin 3' UTR (677 bp) + pBSSA-AR plasmidic sequence (110 bp)

1 acaattcgattatatttatactggactatttttacatctgttcggttattttcacatttatttttctatatatatcttataaacgttttaaaacccatgt
101 aatttttgttaagctgtaatataaaagacgtcctaacaaacttcttttattactgaatttcctttaattataataaataacaagttttaaaataaattca
201 ggcaattaaggcgctcctgaggtactaaaattaatgtaaacatttaaaattaacttggatggtcttaagtactgtactcgtgatttgttatactttatt
301 attagaaaagtcgtctattaactttttgttccttaatttacttgattaaattgtcgcttaatttatcaaatcaggttttgcgcgttattttagagaaaaa
401 cttattagaaaaatgaataagcaaagtttaggctaacatgtttttttattattttaaatagttcaagtcaatgacgtataaaatgcatttgcaaaaaatt
501 ttaagtaaccctataaacttagcaatagtagatactggatgcaagcattcagtagcagcattgcatatctgctgtctttacgtacaaataacagcaaaaa
601 tggacctttattggcttcacatcgtcgtaaaacatgtgttattggacttgtcacaaatgtgttaagtatacagagct 677
1 tagctcttgatgttgatcactagttctagagcggccgccaccgcggtggagctcggcgcgccggtaccgggccccccctcgaggtcgacggtatcgataa
101 gcttgatatc

Hydra Sp5 promoter (2'968 bp) + Sp5 5'UTR (201 bp) + <u>Sp5 coding sequence</u> (27 bp) + MCS (20 bp)

-2968 ctagttctaatttagctctattacgttcgcaaagttgacaaagtcgcaaattttttttcttttttcaaaagacctcccattcattttaataaagactggttc
-2868 taattttgctttatcacgcttataaaaattgacaaagtcgcaagtactttgtttttttaaaagacctcccattcattgagataaatactagttattttcat
-2768 gtatcattgaaatagtaaacaattcattctagttttttatttgtctagggcagtatttcacaccttccacaagtgcgaaacgttttatttacttgattgag
-2668 taaattaatttaaaataaaataaaaaaataagaacttaatgtgaaaaaaaaaacaaaacaaaaacaataaaaaaaaaaacaatgtaaaatatttctcata
-2568 gctgttgaaatattaaaaagacggaaggaaaaatataacggcaaagctaaattcttttctgcgtattgcTTTTTATctctttattgtctgtttgttgctt
-2468 ttcactgcattctattttgtgcttaataaatctcaatcgattttaaggaatgactaggatgtttcattttgtatatatcaataactgaaatattaaaaat
-2368 CTCCTCAGTGCATCCGttcgttagacaattggggtattaactcaattatttcctgaatataaactcaacaagtaaaaaagtttcatccgtaagcaagca
-2268 agaataacgacacttgtttacatttaagaatttcttaactttatgtaaaaaacaattcttagttaaaaacgaagtaaaagggtttaatttttttgttttt

```
-2168  tagttgaaaacaaattgctaaataaaacttaatttaaaaaaaaaaaaccaaatatttaaaatgataaactggttaaaattaatagattatacaaaccatt
                                                                                            Sp5-BS
-2068  gtaagcatttaaaaacaatttttttttataaaaacaacaacaaaaaaaatttcacctaattactCCGCCTttgttaataaaacctccgttttacagttaa
-1968  aagtaatgtatgaaccgtaatccctattacaaaagaaatggtatattgtttataaagacgttttgatgattgtagcttattttatattttgttgctttt
-1868  gtttgttttccttgttgttgtttttaatttgattagttatattcttacggccttacgaccacgtttgctgttttaaatcgcgagcgttgctttgacttta
-1768  cacgaagcctctaaaacaaacataaagagaattcatcgaaagtaaaaaaaatcatgctgaccttcggtgatccgtaattgaaattgatatatattttctc
-1668  cctattttgacatataaatggttaaagttaatcttttttattaaactcaatcaatttcaacaaaataagtgtcaagtttactgcttatttcaagtaacaaa
-1568  taagtcttattgtaaataaagttattgtttaaaagggattgaaacttcaatcattattgtaataaagaagaatttcatgtaagatttgtatttattaaaa
-1468  ttaaataaactaaatgaaacaaaagcgctacgtcaaatttatattttgattattaagaggaattttttttacctaatgtaaaactactgtaaaaatcgact
-1368  gaatcaataaggtcagagagactaggtcagcgagtttggatcattaaaatcgataacaataattaacgatatagtttataatgataggaaacttacactt
                                                                                                  Sp5-BS
-1268  gacatttaaatgggaagtcctgaggctataacgttcgtttgtcgtgggtagataagccaattgacaaaaccatcatcttatatttttatggGCGCCAaat
                         TCF-BS
-1168  gtttatcatgtttaattttCTTTTATataaatgataaaaacatttaaccacaaattatttttttttatctccaaatgaaatcaagaacttttaagtcataaa
                  Sp5-BS
-1068  aagtggcgacaGCGCCAgtgataatcatagacaagtgtacacattagcttatcaaaaagtacgctagagtaaagcttattgttttgttttggctaggata
                                      TCF-BS
 -968  tatccttctcgttaaaataatttgtcctaCTTTTATatacgatattcatattttaggtttcttgtttcgatatatatatatatttcgttatgtttgtat
 -868  gtatatatgtgtttgtttgtatgtgtatgtaaaaatatgaaatatacttttttgcaaatctttgtagaagtttaatataaataaagtatcaagtttaaaaatt
        TCF-BS
 -768  cCTTCGATattttaaagcttcaatttggttgggcgtagacacattagtaattgcggtaaagatcagtaagaattctaaatagacgttaattttaaAACC
         Zic-BS    TCF-BS
 -668  TGGCCTGCCCCTTGATtatttaatttgaaattttaagctgtctccatttcaaccacagtatcaatGGGTCGGCaaaaaaagaaagattgaacgttttat
 -568  caattttaccaacgaaaaacacttaacaaatattgtagtactttttttaagtttaaatgtttttttgtaaaactgttatttttaaaataaacctttttacttc
                                                                                            Zic-BS
 -468  ttttttttttttttgaaatcgttttaaactgatatttaataaaagcttaaatataaatgtggtaaagttcgtaaaaccaatgaaGGCAGGTGCCGGCat
                                                                                        TCF-BS
 -368  agatgaaagtgaaagaacaattttttttttattgaacttcacatttactgtggattgtcggaatgttttactattaagttgATTTGAAGTcaaaaacaaaa
                                                                              TSS3 -194  (H. oligactis)
 -268  taacaaaatcaaccaatgaacttccttagaaattgtttaatcataaaccaatcaaatagcgtttTATAtctttttAaccaataagataacaattttattgt
                      TSS2 -151  (Hm105)          Sp5-BS1                  Sp5-BS2
 -168  tttgtcggcatcttaagAtattaaaagttaatatcttttCCGCCTtacgtattctgtttatcaCCGCCTcttagaccatcccatttgtacgtaaacagag  PP4/PP5
                      Sp5-BS3             Sp5-BS4               TCF-BS         TSS1 (H. AEP)          Sp5-BS5
  -68  aaaatatgatcgcaacGCGCCAtttctcagtcagAGGCGTgacattaacccttATCAAAGaagccgaAgctatttaagataagaTGGCGGtttcttctg  PP4/PP5
  +33  atgtctgaatgaattgtttcggttttttttgaagtaaggataacgttaagaacgatttgaaatgcccagctgatttaaatagaattgaagtagaaagcat
                                                                                  +202
 +133  tcagttaaactttctactttaaagtgcaaagacgttcataagaaatacatttcagtaaaattaagtaaaATGTCACCTCCAAGTCGTGTTCCAA  +227
                                                                                  M   S   P   P   S   R   V   P
       CTGCAGCACCCGGGGAAAAA
```

eGFP coding sequence (717 bp) ***+ MCS (14 bp)***

```
   1.ATGAGTAAAGGAGAAGAACTTTTCACTGGAGTTGTCCCAATTCTTGTTGAATTAGATGGTGATGTTAATGGGCACAAATTTTCTGTCAGTGGAGAGGGTG
 101 AAGGTGATGCAACATACGGAAAACTTACCCTTAAATTTATTTGCACTACTGGAAAACTACCTGTTCCATGGCCAACACTTGTCACTACTTTCTGTTATGG
 201 TGTTCAATGCTTTTCAAGATACCCAGATCATATGAAACGGCATGACTTTTTCAAGAGTGCCATGCCCGAAGGTTATGTACAGGAAAGAACTATATTTTTC
 301 AAAGATGACGGGAACTACAAGACACGTGCTGAAGTCAAGTTTGAAGGTGATACCCTTGTTAATAGAATCGAGTTAAAAGGTATTGATTTTAAAGAAGATG
 401 GAAACATTCTTGGACACAAATTGGAATACAACTATAACTCACACAATGTATACATCATGGCAGACAAACAAAAGAATGGAATCAAAGTTAACTTCAAAAT
 501 TAGACACAACATTGAAGATGGAAGCGTTCAACTAGCAGACCATTATCAACAAAATACTCCAATTGGCGATGGCCCTGTCCTTTTACCAGACAACCATTAC
 601 CTGTCCACACAATCTGCCCTTTCGAAAGATCCCAACGAAAAGAGAGACCACATGGTCCTTCTTGAGTTTGTAACAGCTGCTGGGATTACACATGGCATGG
 701 ATGAACTATACAAATAG    CATTCGTAGAATTC
```

Actin 3' UTR (677 bp)

```
   1 acaattcgattatatttatactggactatttttacatctgttcggttattttcacatttatttttctatatatatcttataaacgttttaaaacccatgt
 101 aatttttgttaagctgtaatataaaagacgtcctaacaaacttcttttattactgaatttcctttaattataataaataacaagttttaaaataaattca
 201 ggcaattaaggcgctcctgaggtactaaaattaatgtaaacatttaaaattaacttggatggtcttaagtactgtactcgtgattttgttatactttatt
 301 attagaaaagtcgtctattaacttttttgttccttaatttacttgattaaattgtcgcttaatttatcaaatcaggttttgcgcgttattttagagaaaaa
 401 cttattagaaaaatgaataagcaaagtttaggctaacatgtttttttattattttaaatagttcaagtcaatgacgtataaaatgcatttgcaaaaaatt
 501 ttaagtaaccctataaacttagcaatagtagatactggatgcaagcattcagtagcagcattgcatatctgctgtctttacgtacaaataacagcaaaaa
 601 tggacctttattggcttcacatcgtcgtaaaacatgtgttattggacttgtcacaaatgtgttaagtatacagagct     677
```

pBSSA-AR plasmidic sequence (713 bp)

```
     1........10........20........30........40........50........60........70........80........90........1
   1 tagctcttgatgttgatcactagagcggccgccaccgcggtggagctcggcgcgccagctccaattcgccctatagtgagtcgtattacgcgcgctcact
 101 ggccgtcgttttacaacgtcgtgactgggaaaaccctggcgttacccaacttaatcgccttgcagcacatccccctttcgccagctggcgtaatagcgaa
 201 gaggcccgcaccgatcgcccttcccaacagttgcgcagcctgaatggcgaatggaaattgtaagcgttaatattttgttaaaattcgcgttaaatttttg
 301 ttaaatcagctcattttttaaccaataggccgaaatcggcaaaatcccttataaatcaaaagaatagaccgagatagggttgagtgttgttccagtttgg
 401 aacaagagtccactattaaagaacgtggactccaacgtcaaagggcgaaaaaccgtctatcagggcgatggcccactacgtgaaccatcaccctaatcaa
 501 gtttttttggggtcgaggtgccgtaaagcactaaatcggaaccctaaagggagcccccgatttagagcttgacggggaaagccggcgaacgtggcgagaaa
 601 ggaagggaagaaagcgaaaggagcgggcgctagggcgctggcaagtgtagcggtcacgctgcgcgtaaccaccacacccgccgcgcttaatgcgccgcta
 701 cagggcgcgtcag      713
```

Figure S2. Map and sequences of the *HyActin-1388*:mCherry_*HySp5-3169*:GFP construct (10'533 bp)

The *Sp5* upstream sequences are composed of 201 bp 5' untranslated (UTR) and 2968 bp promoter sequences. The *HyActin-1388*:mCherry_*HySp5-3169*:GFP construct was further named *HySp5-3169*:GFP construct.

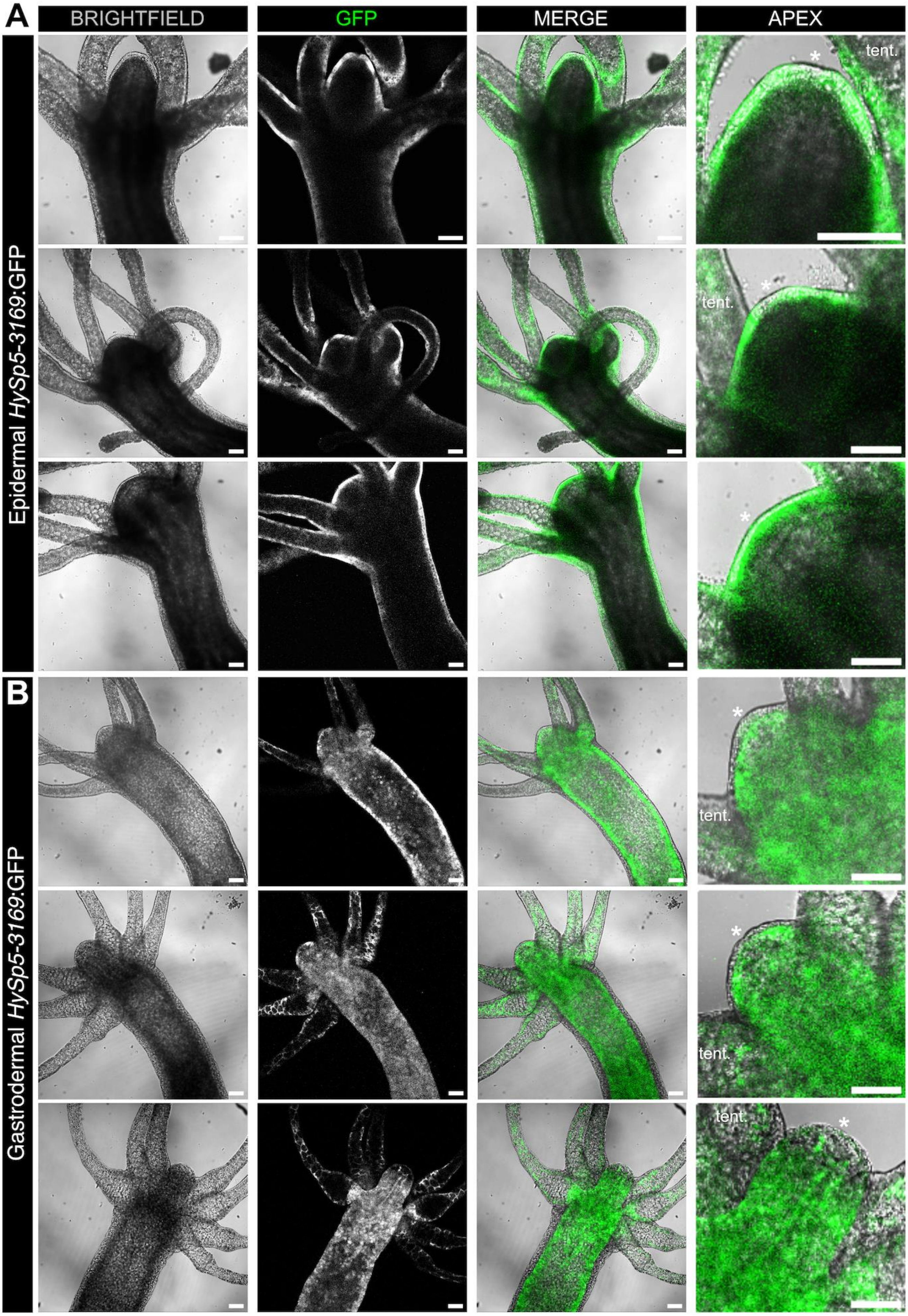
A
BRIGHTFIELD
GFP
MERGE
APEX
Epidermal HySp5-3169:GFP
tent.
B
Gastrodermal HySp5-3169:GFP

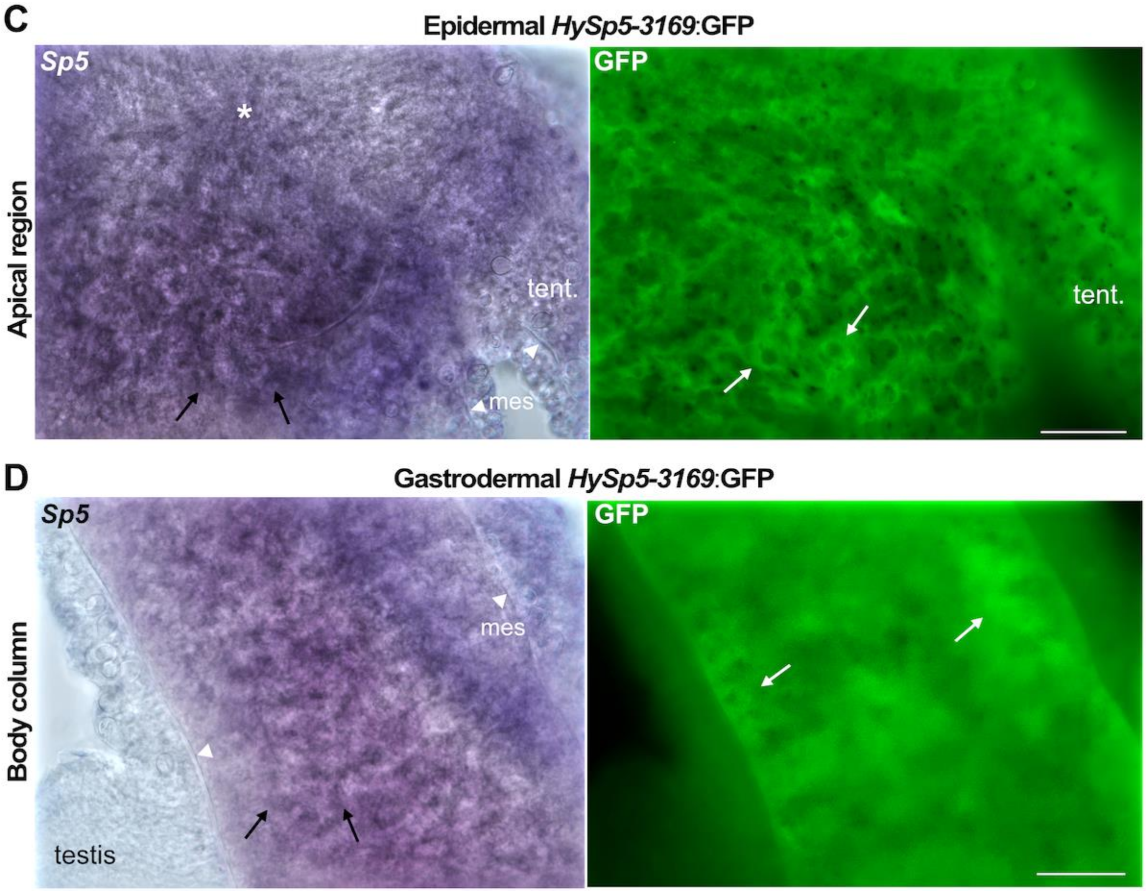

Figure S3. Epidermal and gastrodermal GFP fluorescence in intact *HySp5-3169*:GFP transgenic animals
(A, B) Optical sections of live transgenic animals constitutively expressing *HySp5*-3169:GFP either in the outer layer named epidermis (A) or in the inner layer named gastrodermis (B). Brightfield and eGFP (green) channels are shown, and the most apical region of each animal is magnified as region-of-interest (APEX) with the mouth opening indicated by an asterisk. Imaging was performed on a spinning disc confocal microscope (Nikon Ti inverted) with bright-field and GFP channels. Scale bar: 100 µm. **(C, D)** Co-detection of *Sp5* transcripts by colorimetric staining after *in situ* hybridization (purple) and GFP protein by immunofluorescence (green). White arrowheads indicate the thin extra-cellular layer named mesoglea (mes) that separates the epidermis from the gastrodermis, white arrows indicate epithelial cells expressing GFP at high levels in the epidermis (C) or gastrodermis (D), black arrows epithelial cells expressing *Sp5* in the epidermis (C) or gastrodermis (D), asterisks the mouth opening; tent.: tentacle. Imaging was performed on a widefield Nikon Eclipse 80i microscope able to transmit light and fluorescence equipped with a 40x objective. Scale bar: 50 µm. **Supplement to Figure 1C, 1D**.

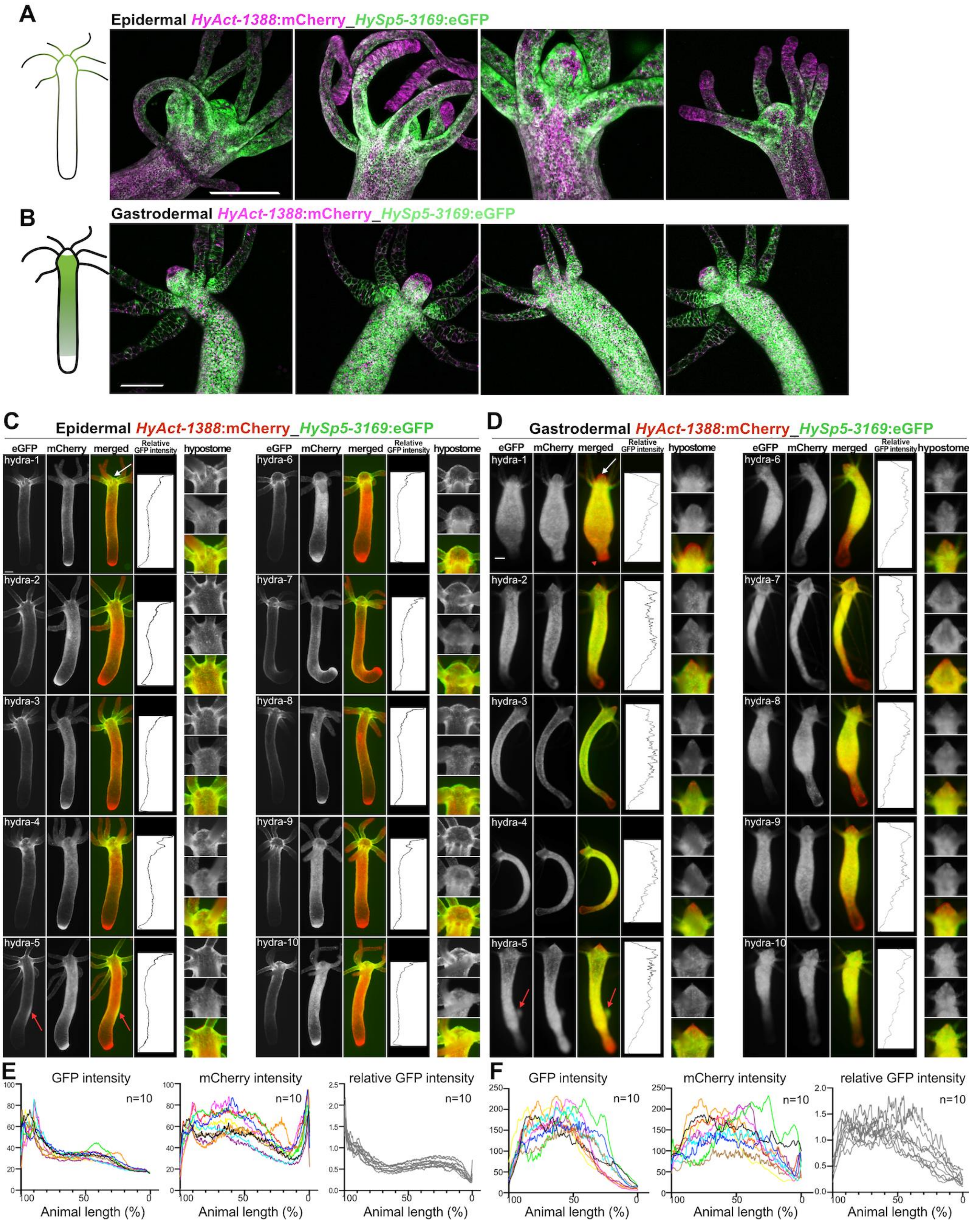

Figure S4. Epidermal and gastrodermal profiles of GFP and mCherry fluorescence in intact *HySp5-3169*:GFP transgenic animals

(A-D) Live imaging of GFP (green) and mCherry (magenta or red) fluorescence in transgenic animals constitutively expressing the *HySp5-3169*:GFP construct in their epidermal (A, C) or gastrodermal (B, D) epithelial cells. In panels C and D, magnified views of the apical region are shown on the right, white arrows indicate the most apical region, red arrowhead the basal region and red arrows the GFP-positive budding regions. **(E, F)** Graphs displaying the GFP (left) and mCherry (middle) fluorescence intensities along the apical (100%) to basal (0%) axis of 10 animals shown either in panel C or in panel D. The relative GFP intensity (right panel) is calculated from the ratio between the GFP and mCherry fluorescence. Scale bars: 250 µm in all panels. **Supplement to Figure 1G, 1H, 1K.**

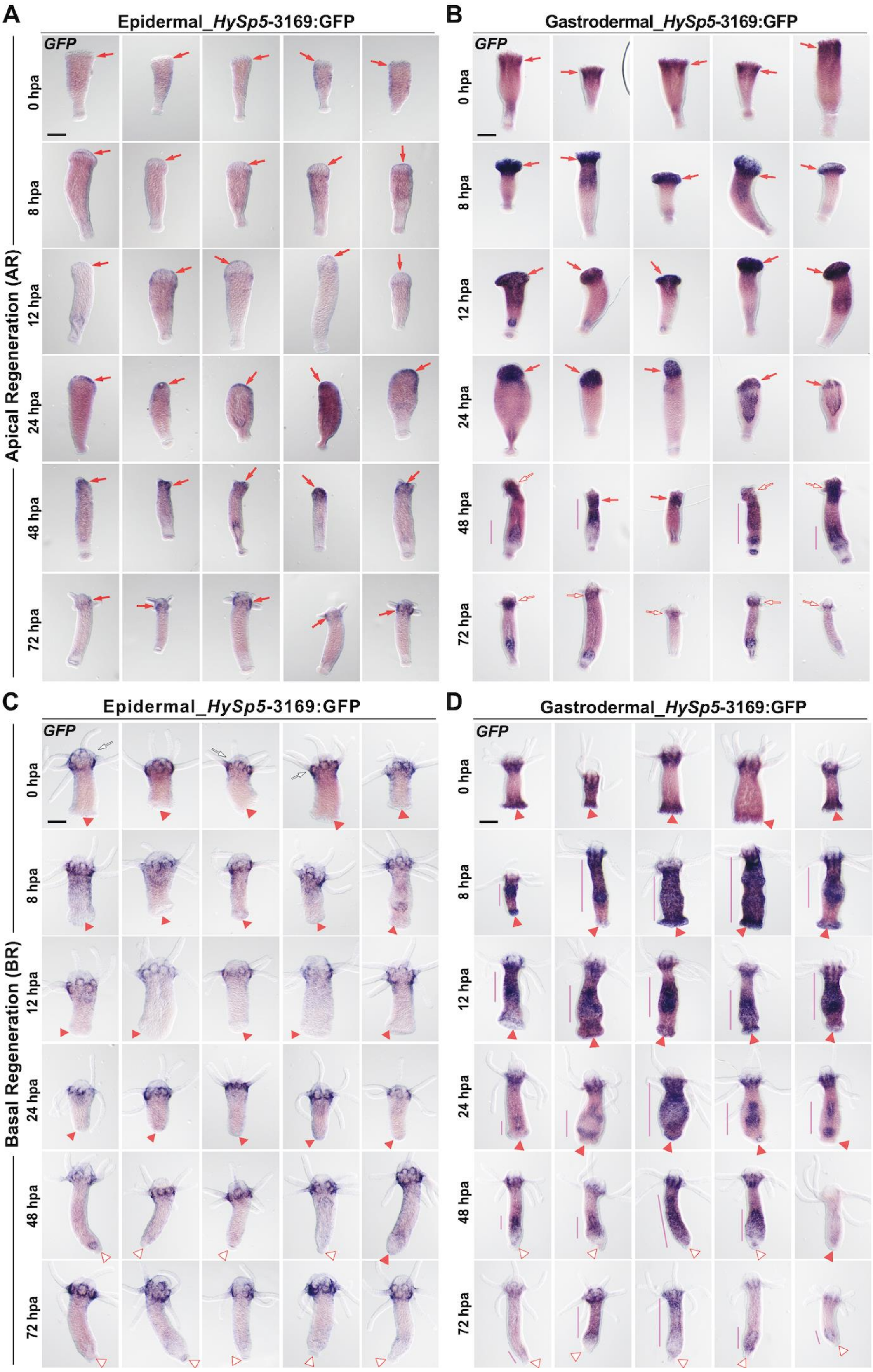

Figure S5. Layer-specific *GFP* expression in regenerating *HySp5-3169*:GFP transgenic animals

GFP expression in lower halves that are apical-regenerating (AR) **(A, B)** and upper halves that are basal-regenerating (BR) **(C, D)** from epidermal_ **(A, C)** and gastrodermal_ **(B, D)** *HySp5-3169*:GFP transgenic animals taken at 0, 8, 12, 24, 48 and 72 hours post-amputation (hpa). Vertical bars indicate areas of *GFP* expression along the body column. White arrows point to original heads; red arrows to apical-regenerating tips; white arrows outlined red to regenerated heads; white triangles

to original basal discs; red triangles to basal-regenerating tips, and white triangles outlined red to regenerated basal discs. Scale bar: 250 µm. **Supplement to Figure 2A, 2B.**

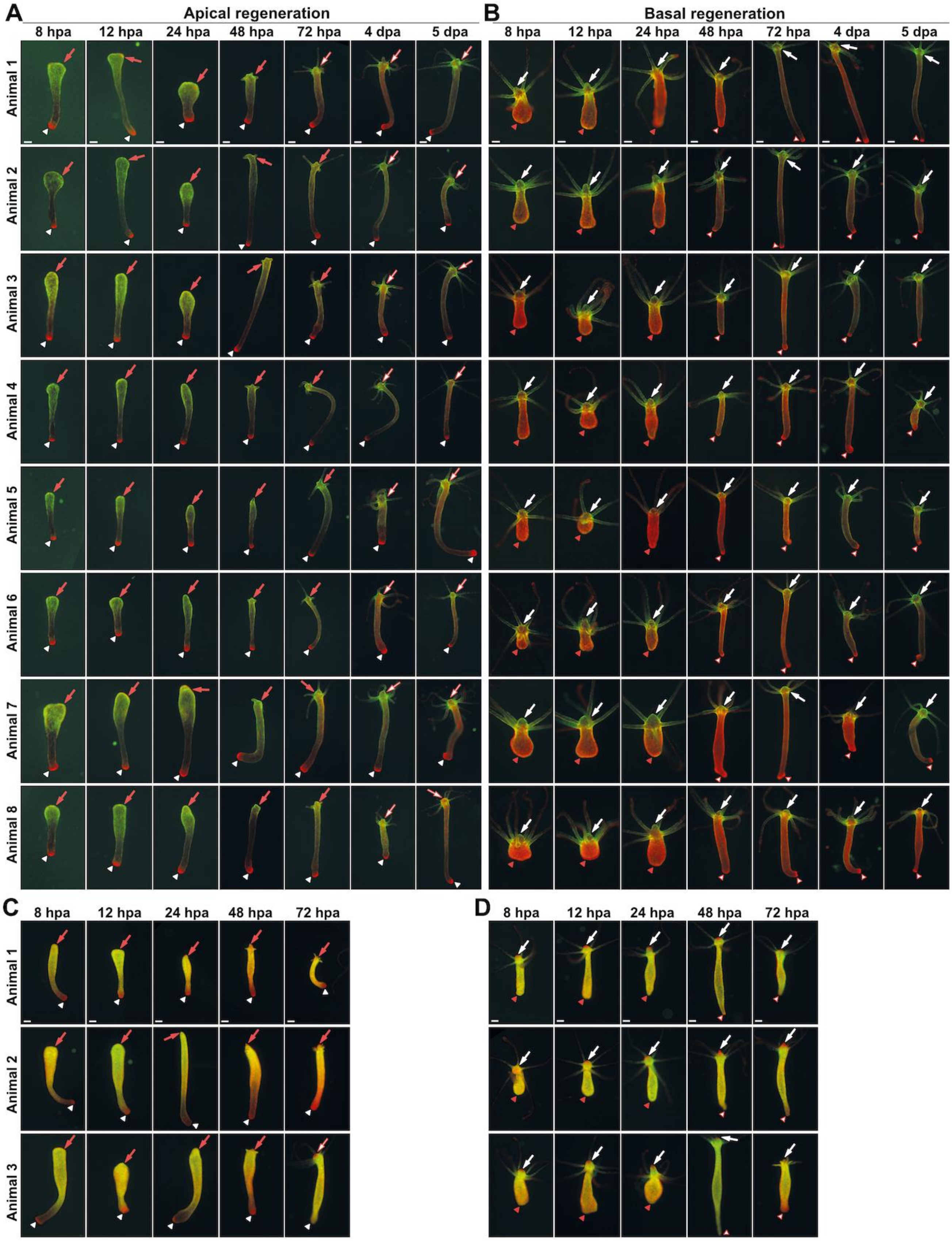

Figure S6. GFP and mCherry fluorescence in apical-regenerating (AR) or basal-regenerating (BR) halves of epidermal (A, B) and gastrodermal (C, D) *HySp5*-3169:GFP transgenic animals

Live imaging of GFP (green) and mCherry (red) fluorescence in apical-regenerating (A, C) and basal-regenerating (B, D) halves of epidermal and gastrodermal *HySp5-3169*:GFP animals pictured at indicated time-points; hpa: hours post-amputation, dpa: days post-amputation. White arrows point to original heads; red arrows to AR tips; white arrows outlined red to regenerated heads; white arrowheads to original basal discs; red arrowheads to BR tips; white arrowheads outlined red to regenerated basal discs. Scale bar: 250 µm. **Supplement to Figure 2C and 2D.**

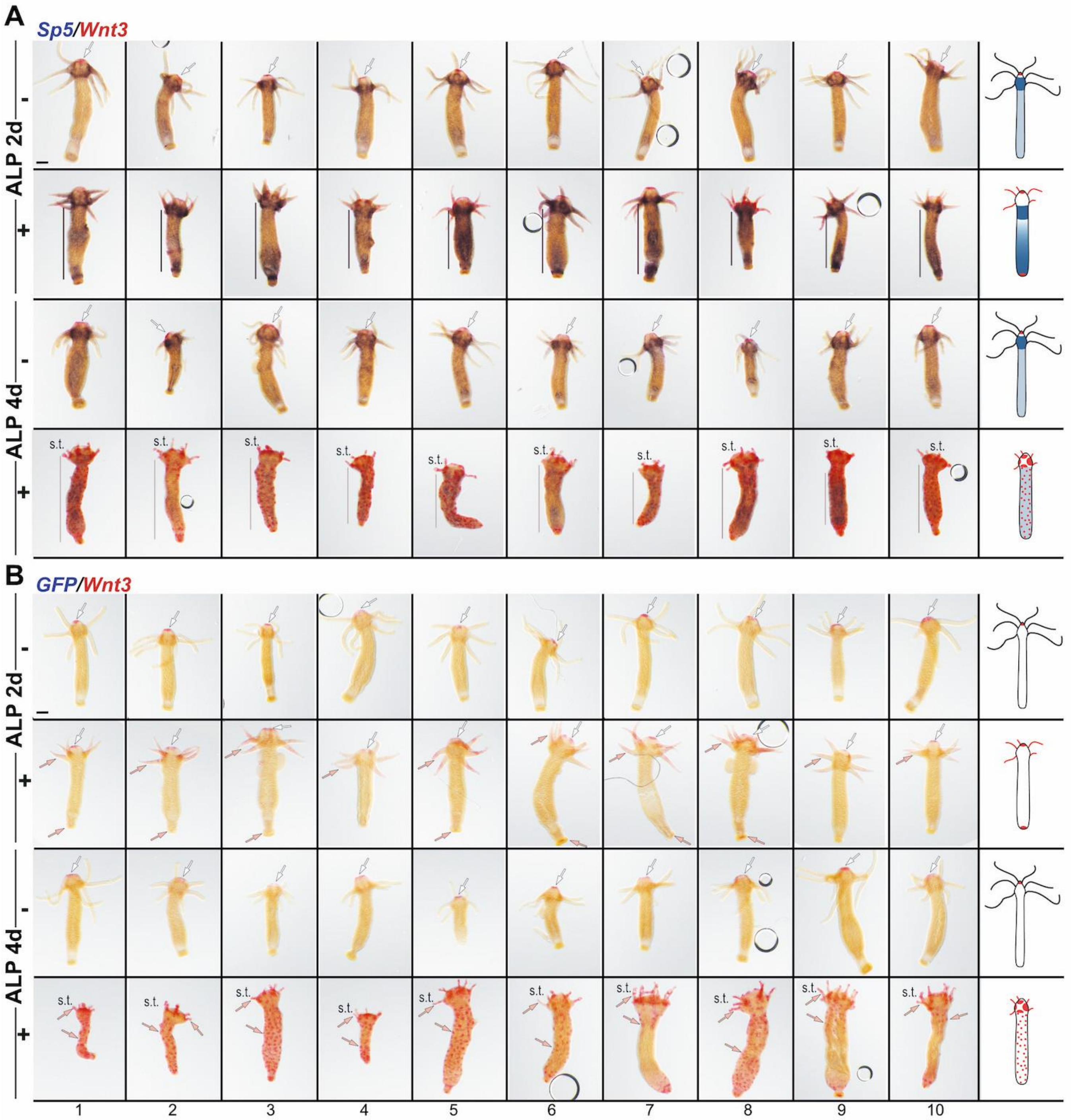

Figure S7. Impact of alsterpaullone (ALP) treatment on *Sp5* and *Wnt3* expression in non-transgenic *Hv_AEP2* animals

Co-detection of *Sp5* (dark blue) and *Wnt3* (red) **(A)**, or of *GFP* (dark blue) and *Wnt3* (red) **(B)** in wild-type *Hv_AEP2* animals left untreated (DMSO) or exposed to alsterpaullone (ALP) for 2 or 4 days. White arrows point to apical *Wnt3* homeostatic expression; salmon arrows to ectopic *Wnt3* expression; vertical bars to *Sp5* expression along the body column; s.t.: short

tentacles. As expected, non-transgenic *Hv_AEP2* animals do not express *GFP*. Schematic views of *Hydra* polyps on the right depict the typical expression profile of each condition. Scale bar: 200 µm. **Supplement to Figure 3B.**

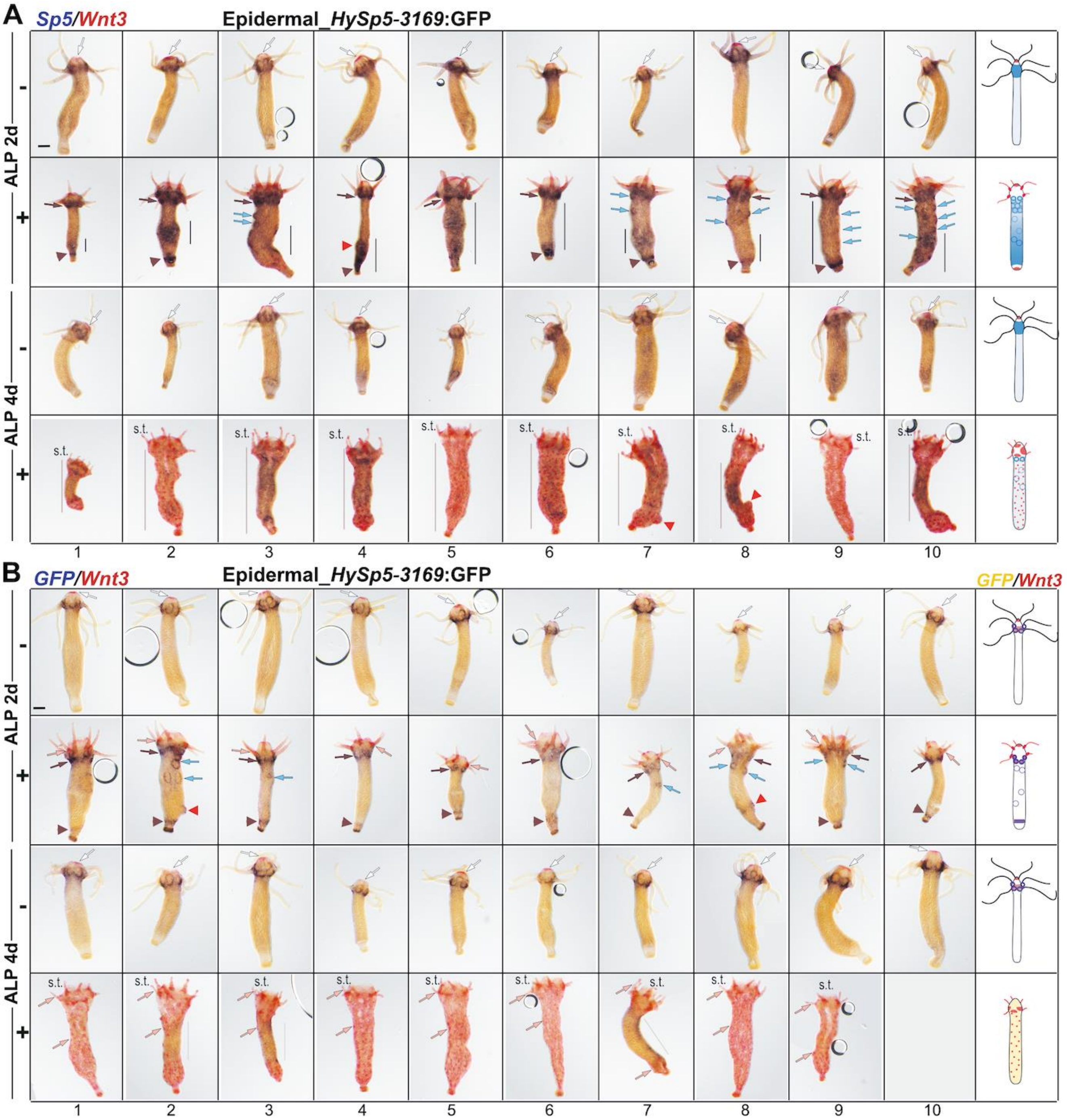

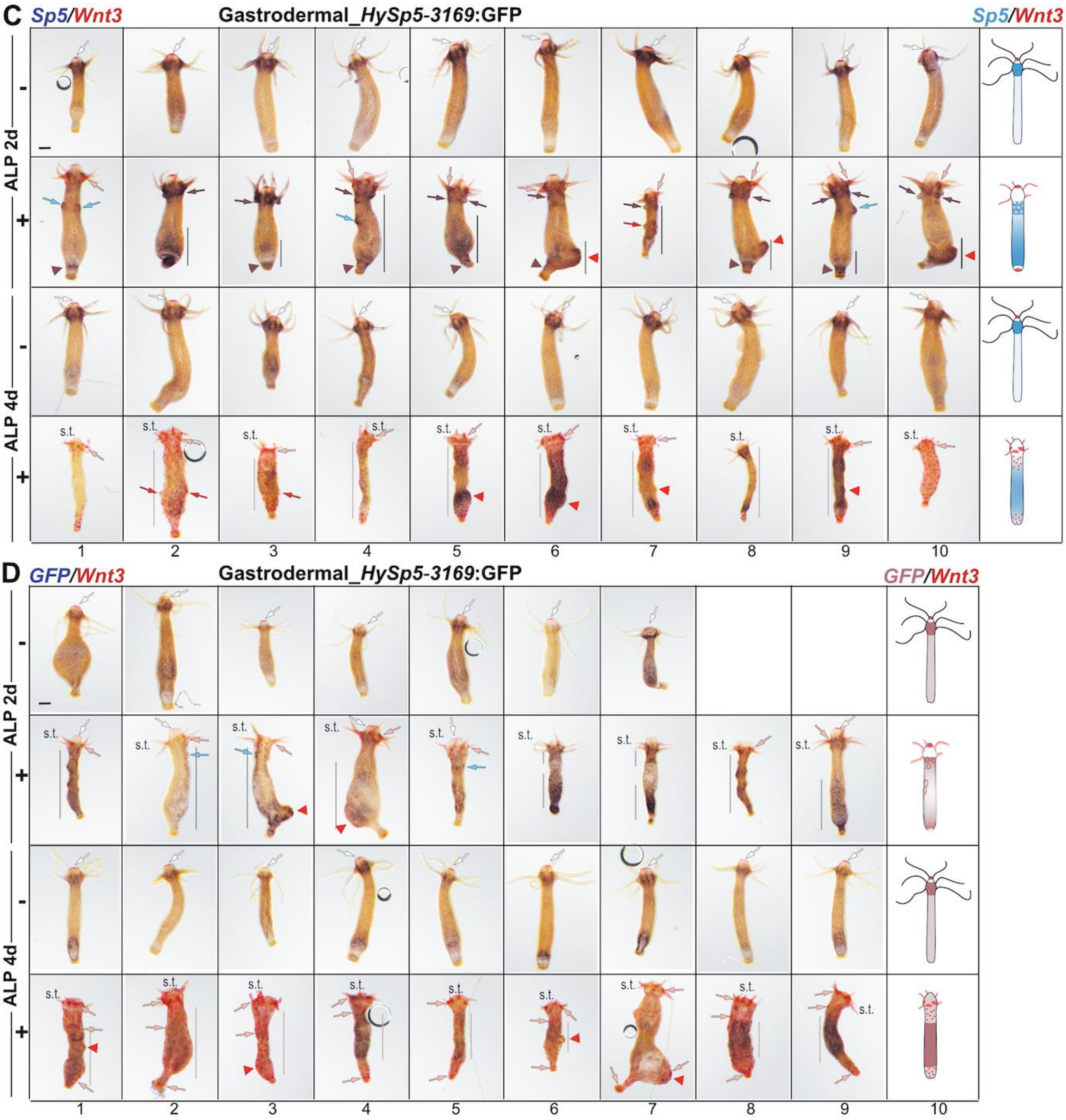

Figure S8. Impact of ALP treatment on *Sp5, GFP* and *Wnt3* expression patterns in epidermal and gastrodermal *HySp5-3169:GFP* transgenic animals.

(A, C) Co-detection of *Sp5* (dark blue) and *Wnt3* (red) expression in epidermal (A) or gastrodermal (C) *HySp5-3169:*GFP animals treated with DMSO (-) or ALP (+) for 2 or 4 days. **(B, D)** Co-detection of *GFP* (dark blue) and *Wnt3* (red) expression in epidermal (B) or gastrodermal (D) *HySp5-3169:*GFP animals treated with DMSO (-) or ALP (+) for 2 or 4 days. In all panels brown arrows point to *Sp5* (A, C) or *GFP* (B, D) expression in the upper body column immediately below the tentacle ring; light blue arrows to spots of *Sp5* (A, C) or *GFP* (B, D) expression in the body column; vertical bars to diffuse *Sp5* or *GFP* expression along the body column; black triangles to *Sp5* (A) or *GFP* (B) basal expression; red triangles indicate bud-like structures growing from the lower body column expressing *Sp5* (A, C) or *GFP* (B, D); white arrows to *Wnt3* expression at the tip of the hypostome; salmon arrows to ectopic *Wnt3* expression in tentacles, along the body column, at basal extremity. Schematic views of *Hydra* polyps on the right depict the typical expression profile of each condition. s.t.: short tentacles; scale bars: 200 µm. Supplement to Figure 3B, 3C.

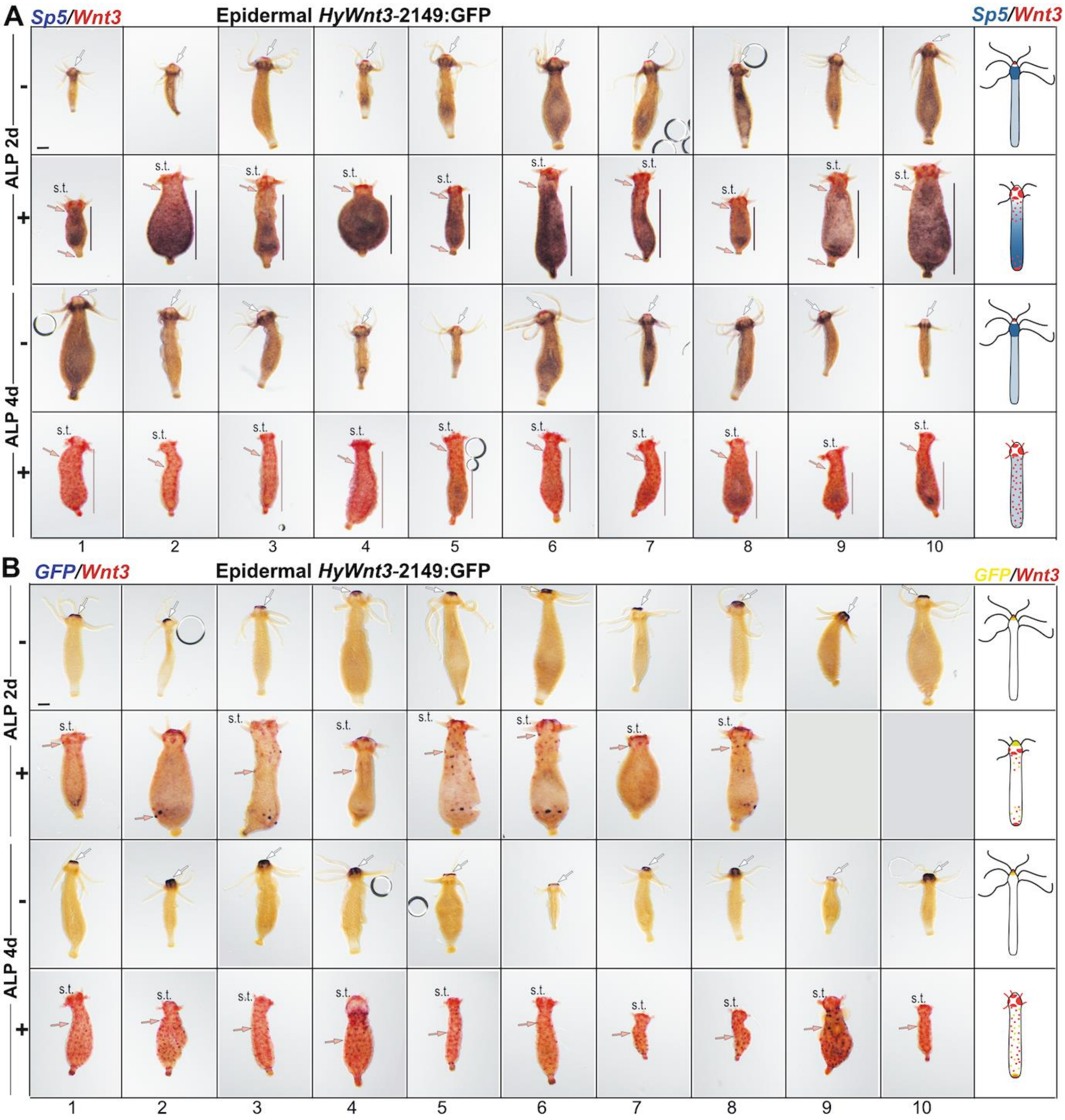
A
Sp5/Wnt3
Epidermal HyWnt3-2149:GFP
Sp5/Wnt3
ALP 2d
ALP 4d
-
+
s.t.
1
2
3
4
5
6
7
8
9
10
B
GFP/Wnt3
Epidermal HyWnt3-2149:GFP
GFP/Wnt3

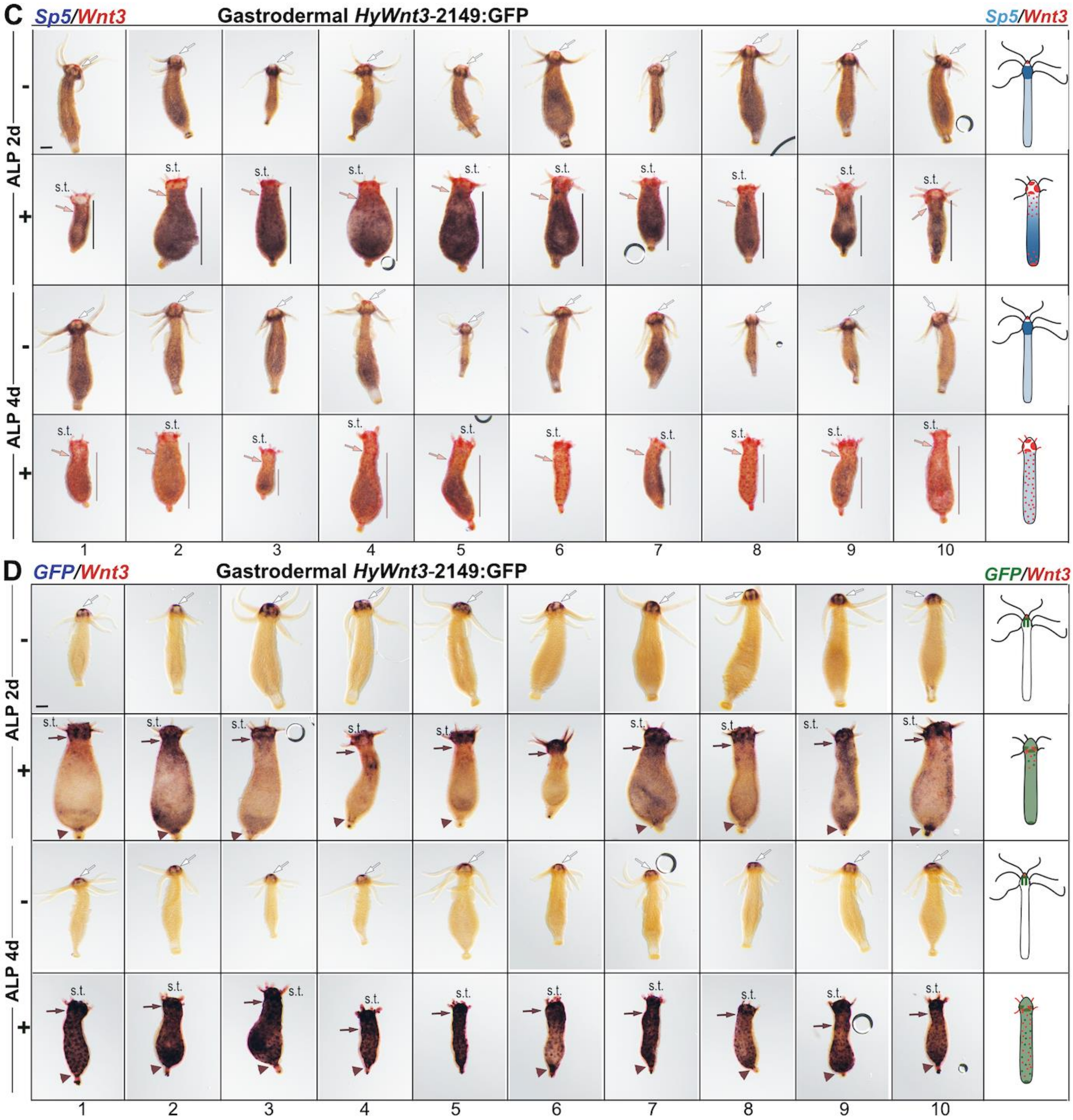

Figure S9. Impact of ALP treatment on *Sp5, GFP* and *Wnt3* expression patterns in epidermal and gastrodermal *HyWnt3-2149*:GFP transgenic animals.

(A, C) Co-detection of *Sp5* (dark blue) and *Wnt3* (red) in epidermal (A) or gastrodermal (C) *HyWnt3-2149:*GFP animals treated with DMSO (-) or ALP (+) for 2 or 4 days. In both panels, vertical bars indicate *Sp5* expression along the body column; white arrows, *Wnt3* expression at the tip of the hypostome; salmon arrows: ectopic *Wnt3* expression in the tentacles, along the body column or at the basal extremity. **(B, D)** Co-detection of *GFP* (dark blue) and *Wnt3* (red) in epidermal (B) or gastrodermal (D) *HyWnt3-2149:*GFP animals treated with DMSO (-) or ALP (+) for 2 or 4 days. In both panels, white arrows indicate *GFP* expression at the tip of the hypostome; brown arrows *GFP* expression at the base of the apical region; salmon arrows ectopic *Wnt3* expression in the body column and brown triangles *GFP* expression at the basal extremity. For each row, a schematic view of the expression profile typical of the experimental condition is shown on the right; s.t.: short tentacles; scale bars: 200 µm. Supplement to Figure 3B, 3C.

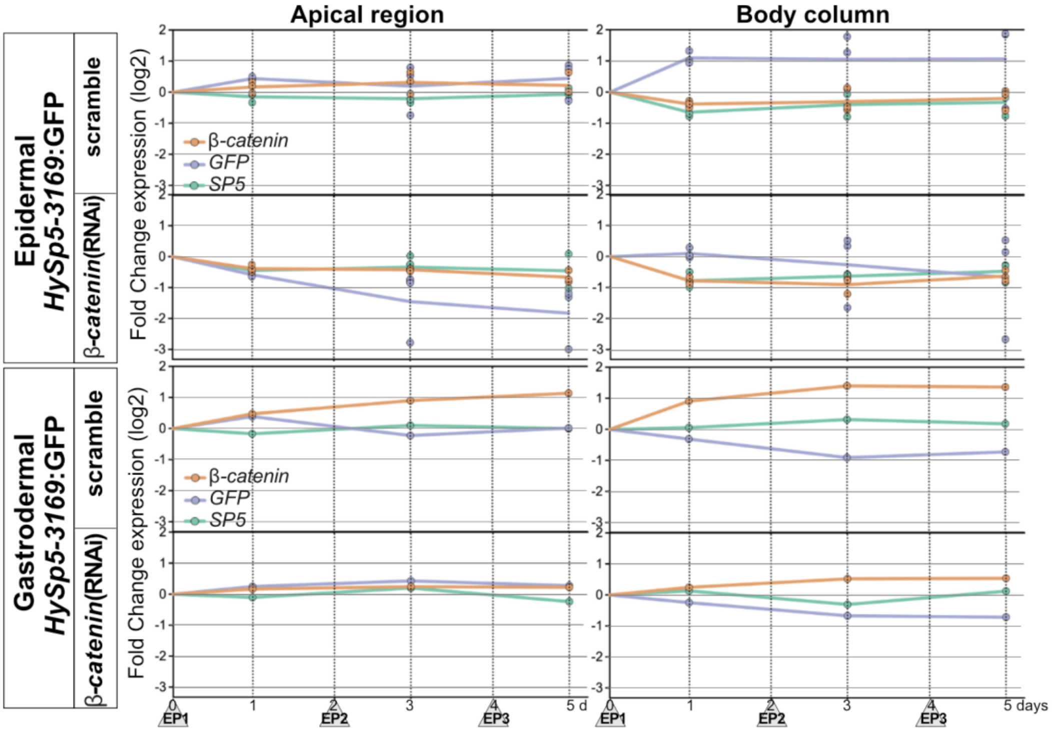

Figure S10. Impact of *β-catenin*(RNAi) on *β-catenin*, *Sp5* and *GFP* transcript levels in epidermal and gastrodermal *HySp5-3169*:GFP transgenic animals

Quantitative-PCR (qPCR) quantification of *β-catenin*, *GFP* and *Sp5* transcripts measured one day after EP1, one day after EP2, or one day after EP3 in *HySp5*-3169:GFP animals exposed to scramble or *β-catenin* siRNAs. Values are expressed as Fold Change (log2) when compared to the value measured in non-electroporated *Hv_AEP2* animals taken at time 0, just before (RNAi) animals are submitted to EP1. Three independent experiments were performed on epidermal *HySp5-3169:GFP* transgenic animals and one experiment on gastrodermal *HySp5-3169:GFP* transgenic animals. Supplement to Figure 4B.

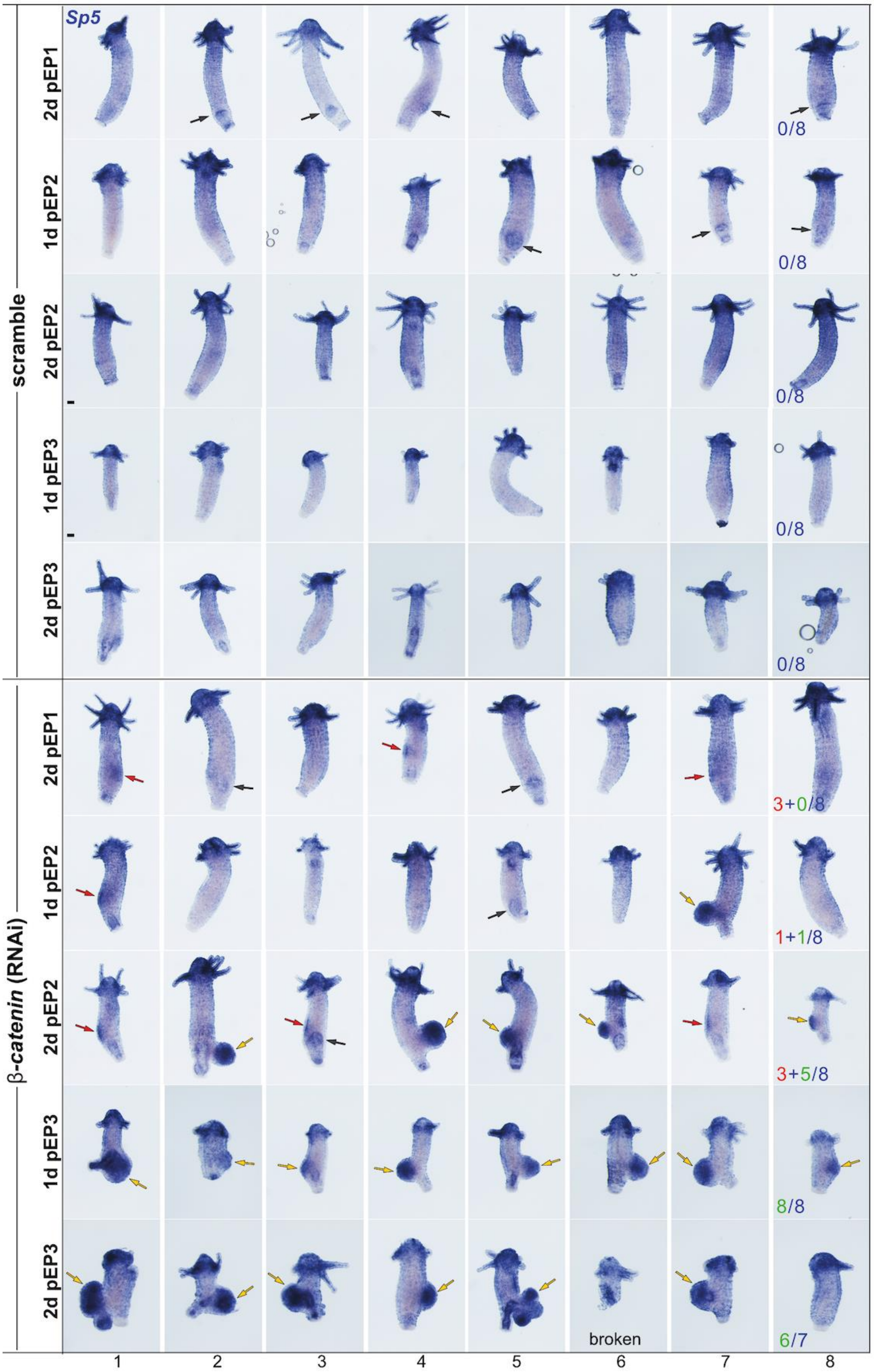

Figure S11. Impact of *β-catenin*(RNAi) on formation of bud-like structures expressing *Sp5* in *Hv-Basel* animals
Sp5 expression in *Hv_Basel* animals electroporated one to three times (EP1, EP2, EP3) with scramble or *β-catenin* siRNAs and fixed as indicated: 2 days post-EP1 (2dpEP1), 1d or 2d post-EP2, 1d or 2d post-EP3. Black arrows point to detached buds, red arrows to *Sp5*-expressing patches along the body column, yellow arrows to *Sp5*-expressing bud-like structures, which become multiple at 2 days post-EP3 (2dpEP3). Scale bars: 200 µm. **Supplement to Figure 4C.**

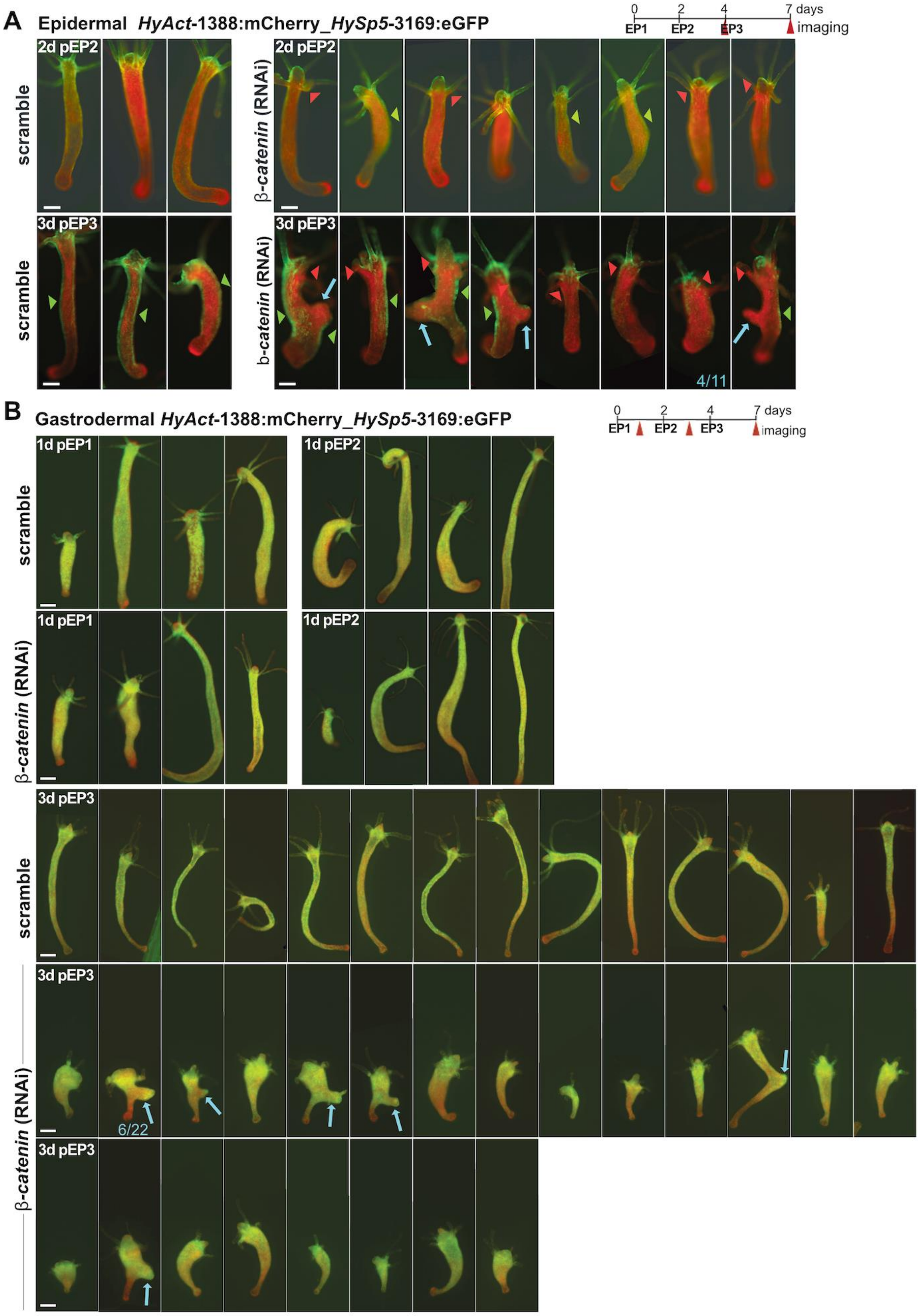

Figure S12. Impact of *β-catenin*(RNAi) on GFP and mCherry fluorescence in epidermal and gastrodermal *HySp5-3169*:GFP transgenic animals after one, two or three siRNA exposures

GFP (green) and mCherry (red) fluorescence in epidermal **(A)** and gastrodermal **(B)** *HySp5*-3169:GFP transgenic animals electroporated with scramble or *β-catenin* siRNAs and pictured live as indicated. Red triangles indicate apical areas where GFP fluorescence is reduced, green arrowheads and green bars areas of ectopic GFP fluorescence along the body column, and blue arrows the bud-like structures that develop at mid-body in *β-catenin* (RNAi) animals, 4/11, in epidermal *HySp5-3169*:GFP animals and all GFP-negative animals (A), 6/22 in gastrodermal *HySp5-3169*:GFP animals and all GFP-positive (B). Supplement to Figure 4C.

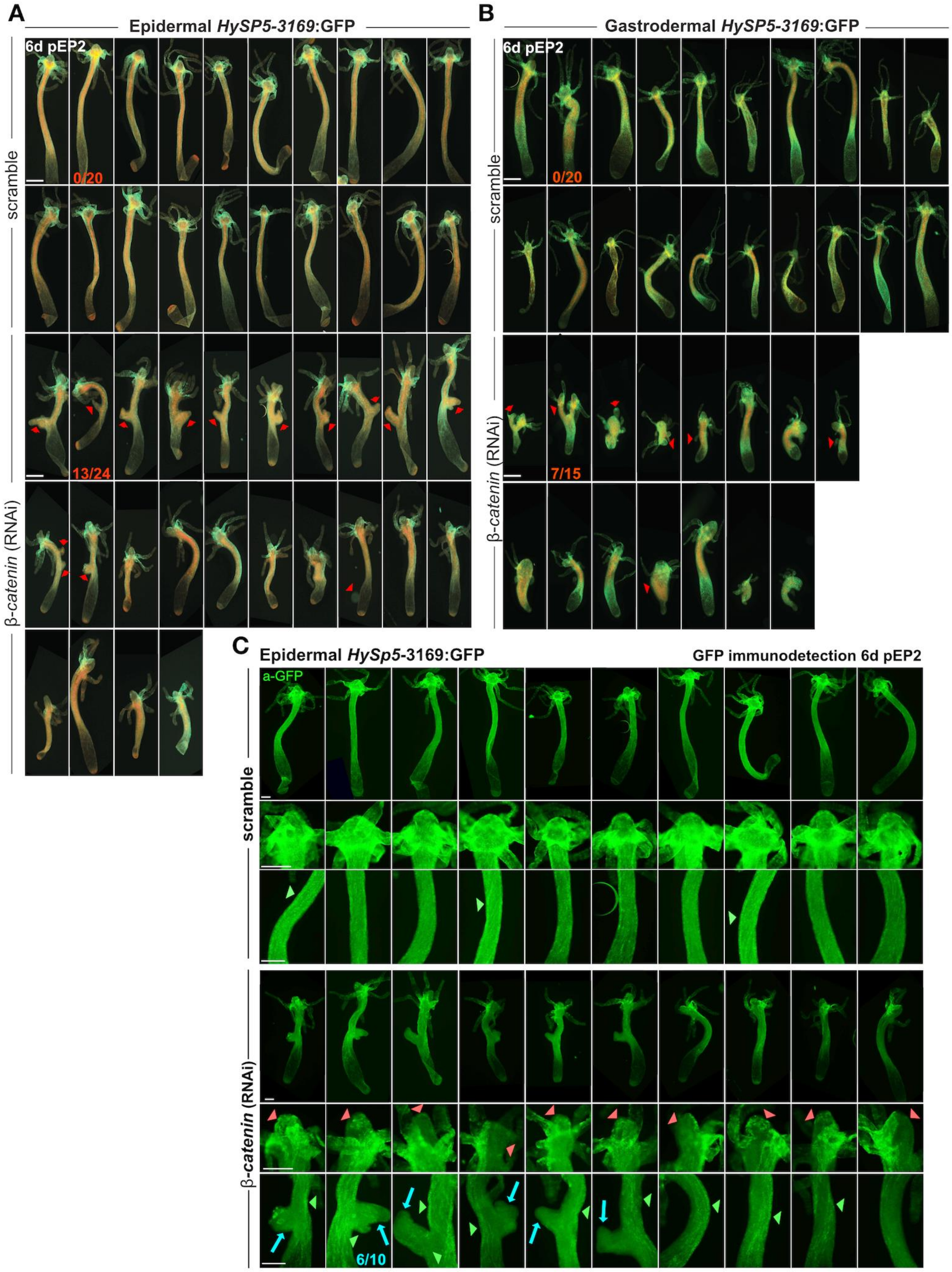

Figure S13. Impact of *β-catenin*(RNAi) on GFP and mCherry immunodetected patterns in epidermal and gastrodermal *HySp5-3169*:GFP transgenic animals six days after the 2nd siRNA exposure

(A, B) GFP (green) and mCherry (red) immunodetected in epidermal (A) and gastrodermal (B) *HySp5*-3169:GFP transgenic animals six days after the second electroporation (6d pEP2) with scramble or *β-catenin* siRNAs. Red arrowheads indicate bud-like structures that develop along the body column. **(C)** GFP immunodetected in epidermal_*HySp5*-3169:GFP transgenic animals 6 days after the 2nd electroporation (6d pEP2) to scramble or *β-catenin* siRNAs. Enlarged views of the apical region and body column are shown for each condition. Note that in *β-catenin* (RNAi) animals GFP is largely reduced in apical regions (red triangles), absent from the bud-like structures (blue arrows) and present in some limited areas along the body column (green triangles). Scale bars: 250 µm. Supplement to Figure 4D.

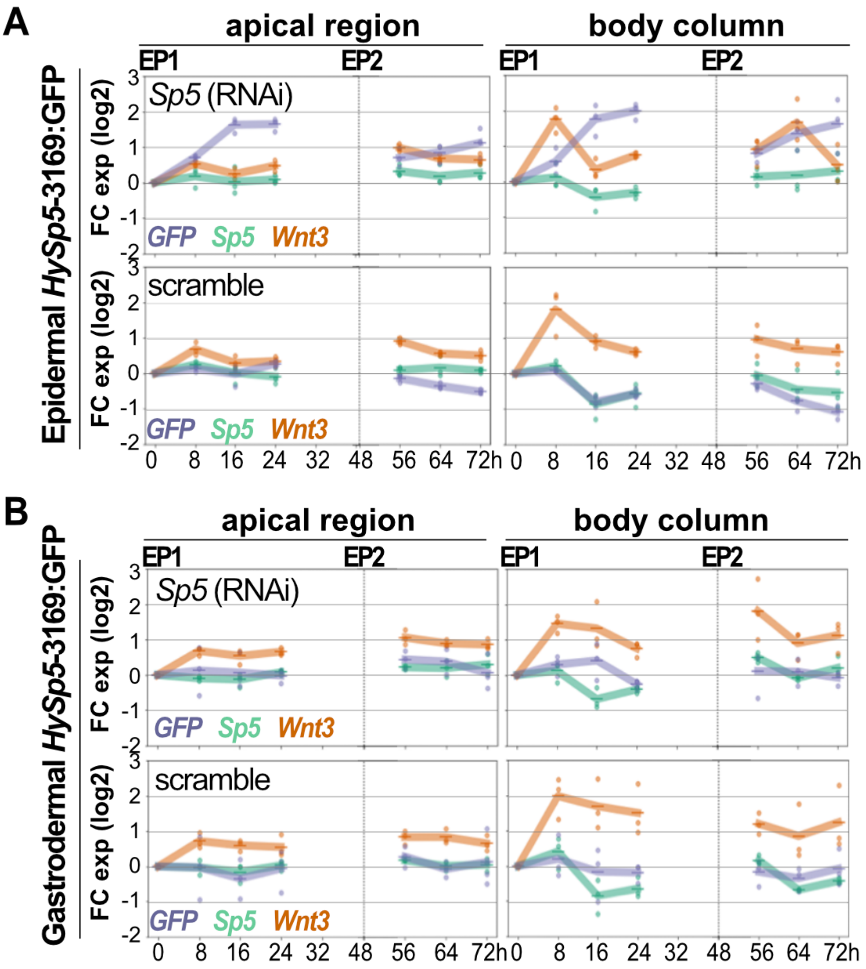

Figure S14. Impact of *Sp5*(RNAi) on *Sp5, GFP* and *Wnt3* transcript levels in epidermal and gastrodermal *HySp5-3169*:GFP transgenic animals

Quantitative PCR analysis of *Sp5, Wnt3* and *GFP* expression levels in the apical (100%-80%, left) and body column (80% - 0%, right) regions of epidermal **(A)** or gastrodermal **(B)** *HyAct1388*:mCherry_*HySp5-3169:*GFP transgenic animals. These animals were electroporated once or twice (EP1, EP2) with scramble or *Sp5* siRNAs and imaged 8, 16, 24 hours post-EP1 and post-EP2 as described in **Figure 5A**. In both panels, the colored lines correspond to the Fold Change values (FC log2) obtained by dividing values measured in *Sp5*(RNAi) or scramble animals by the reference value obtained in non-electroporated *Hv_AEP2* animals (reference at time 0, just before EP1). Note the marked increase in *GFP* expression in the apical region and body column of *Sp5*(RNAi) epidermal *HySp5-3169:*GFP transgenic animals. Supplement to Figure 5A.

A **Epidermal *HyAct-1388*:mCherry_*HySp5-3169*:GFP transgenic *Hydra***

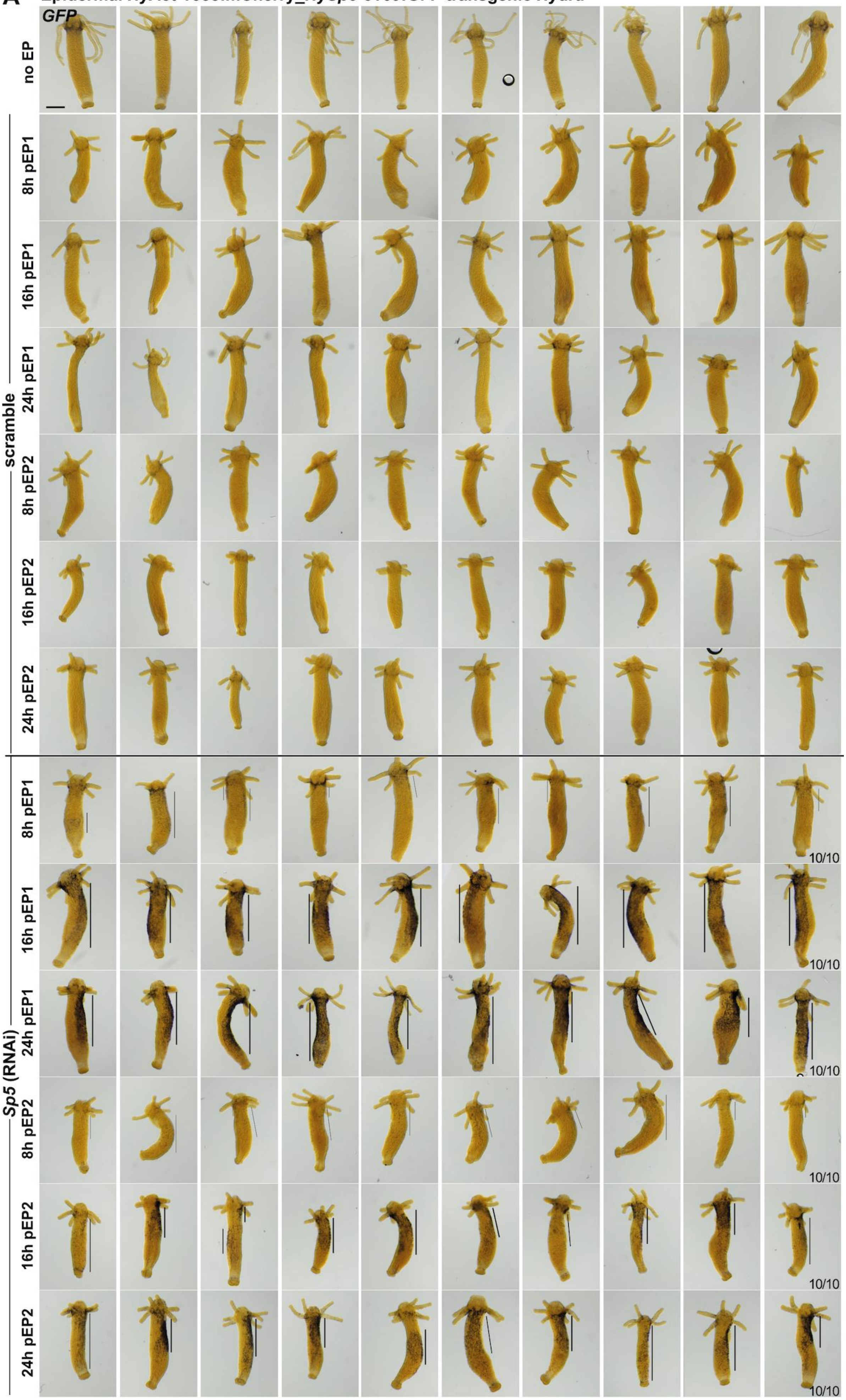

B Gastrodermal *HyAct-1388*:mCherry_*HySp5-3169*:GFP transgenic *Hydra*

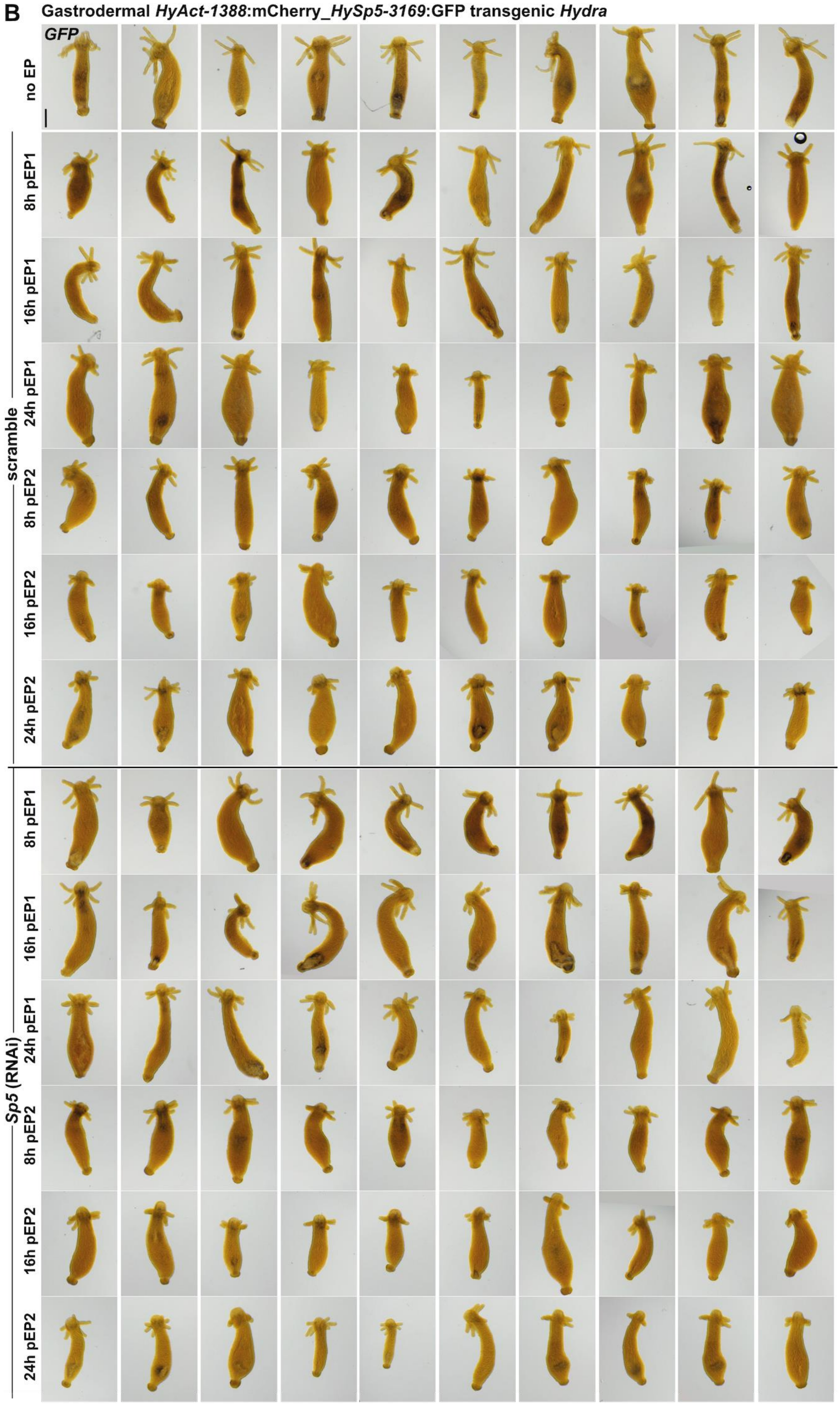

Figure S15. Impact of *Sp5*(RNAi) on *GFP* expression patterns in epidermal and gastrodermal *HySp5-3169*:GFP animals *GFP* expression in *HySp5-3169*:GFP animals either left untreated (wt, topmost row), or electroporated once or twice (EP1, EP2) with scramble or *Sp5* siRNAs and fixed for WM-ISH at 8, 16, 24 hours post-EP1 (pEP1) and 8, 16, 24 hours post-EP2 (pEP2) as described in **Figure 5A**. In epidermal *HySp5-3169*:GFP animals **(A)**, note the ectopic *GFP* expression along the body column (vertical bars). In gastrodermal *HySp5-3169*:eGFP animals **(B)**, no significant modulations can be observed in *Sp5* (RNAi) conditions. Scale bars: 250 µm. **Supplement to Figure 5B.**

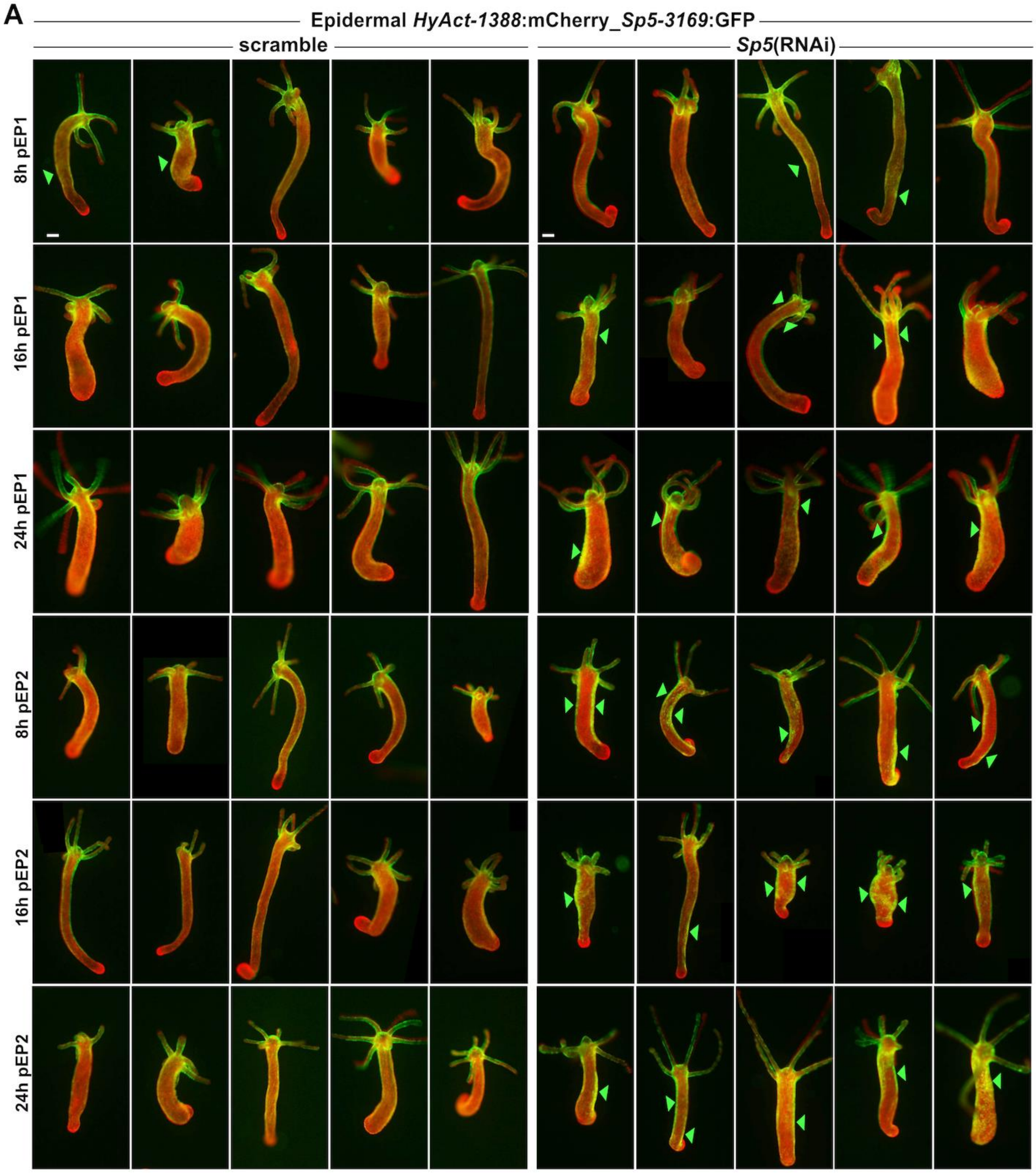
A
Epidermal HyAct-1388:mCherry_Sp5-3169:GFP
scramble
Sp5(RNAi)
8h pEP1
16h pEP1
24h pEP1
8h pEP2
16h pEP2
24h pEP2

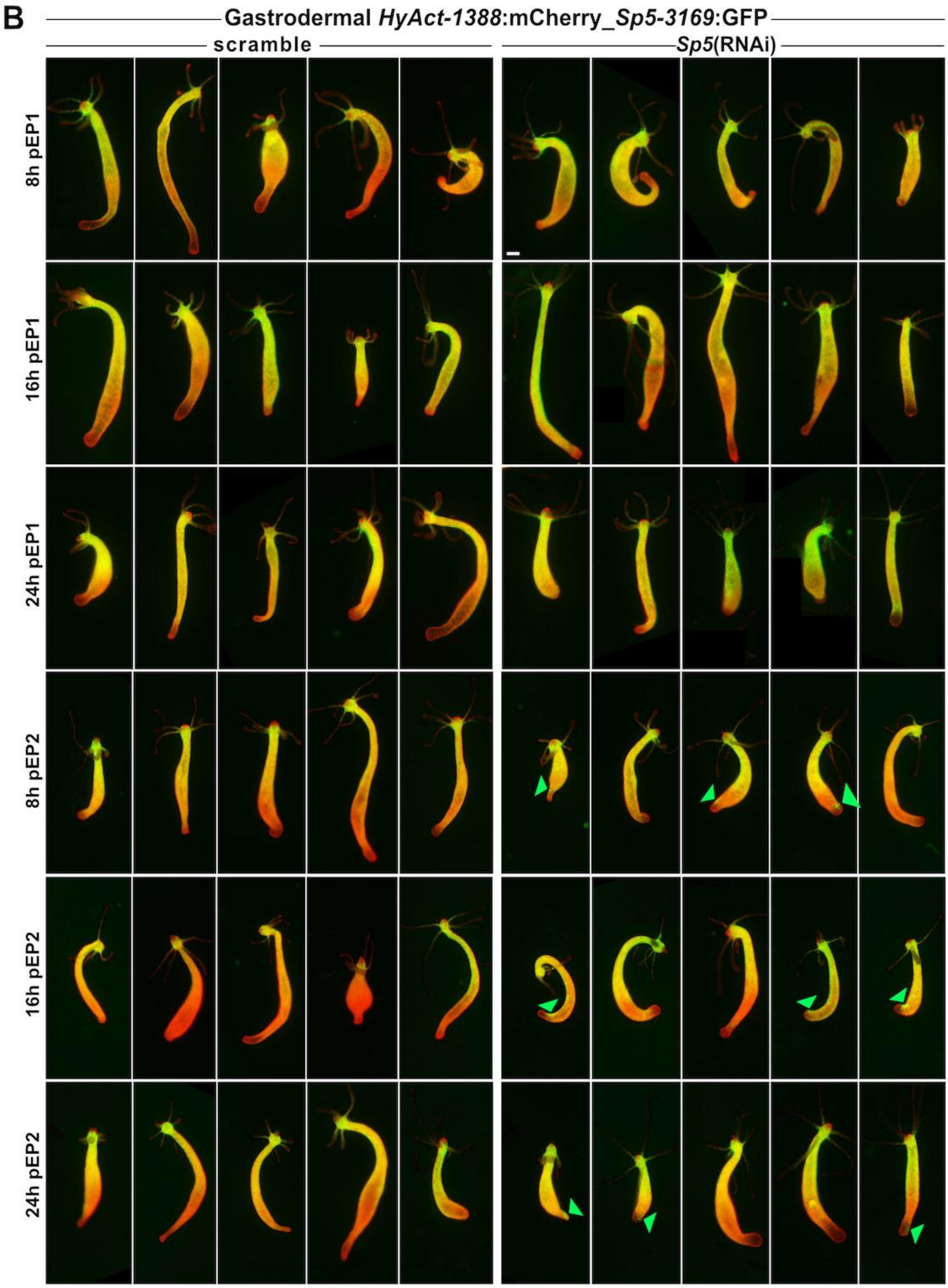

Figure S16. Impact of *Sp5 (RNAi)* on GFP and mCherry fluorescence in epidermal (A) or gastrodermal (B) *HySp5-3169*:GFP transgenic animals at early time-points after one or two siRNA exposures

Animals were electroporated once (EP1) or twice (EP2) with scramble or *Sp5* siRNAs and imaged 8, 16, 24 hours post-EP1 (pEP1) and 8, 16, 24 hours post-EP2 (pEP2) as described in **Figure 5A**. Images from GFP (green) and mCherry (red) merged channels are shown. Green triangles indicate areas of ectopic GFP fluorescence along the body column. Scale bars: 200 µm. **Supplement to Figure 5C.**

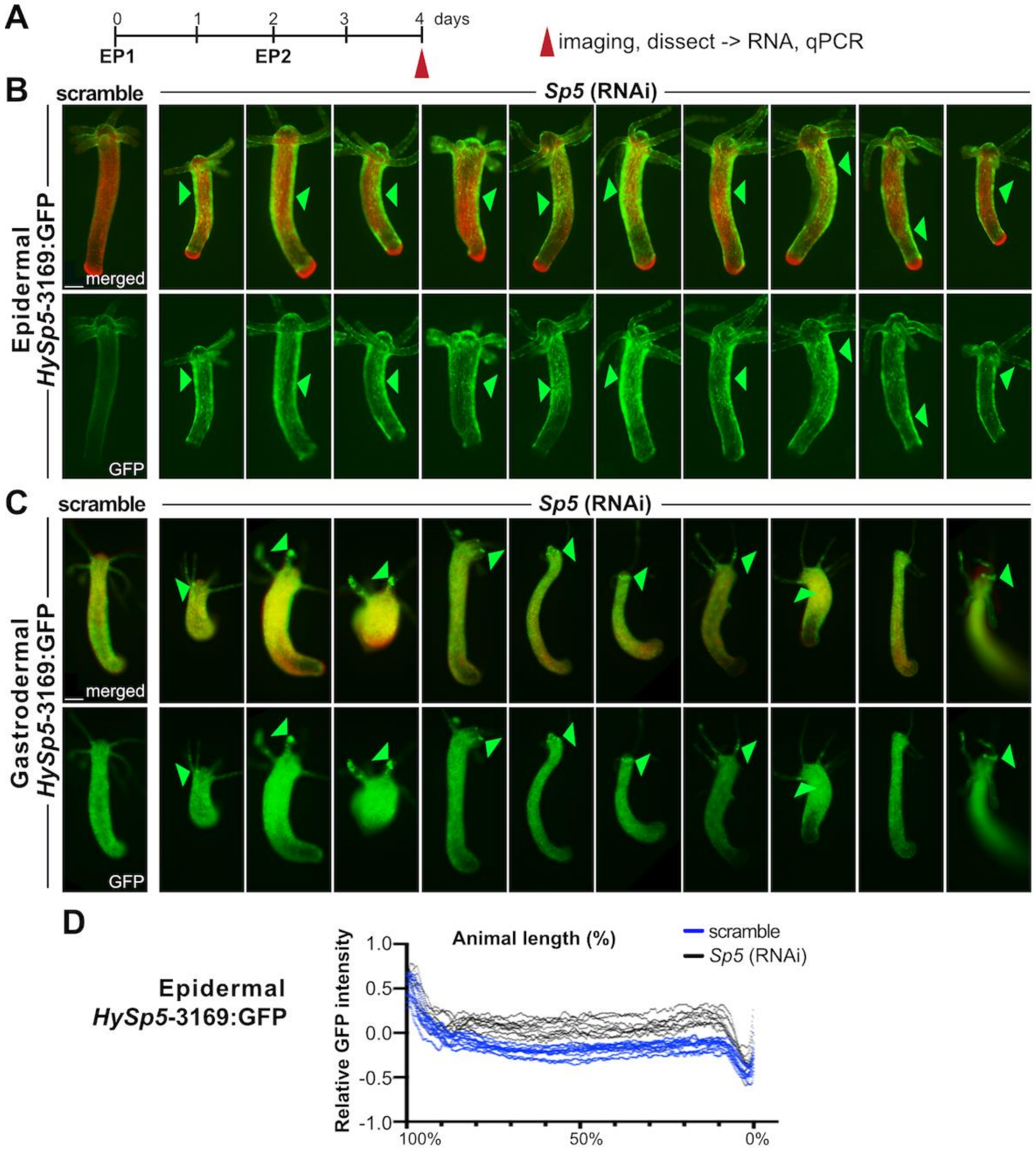

Figure S17. Impact of *Sp5(RNAi*) on GFP and mCherry fluorescence in epidermal and gastrodermal *HySp5-3169*:GFP animals two days after the 2nd siRNA exposure

(A) Schematic view of the procedure where animals are exposed twice to scramble or *Sp5* siRNAs and pictured two days later (2d pEP2). **(B, C)** Live imaging of epidermal (B) and gastrodermal (C) *HySp5*-3169:GFP transgenic animals performed two days after EP2. Green triangles point to ectopic areas of GFP fluorescence, as extended areas along the epidermis, or as gastrodermal spots in the tentacles. Scale bars: 250 µm. (**D)** Measurement two days post-EP2 of the relative GFP intensity in epidermal_*HySp5*-3169:GFP animals exposed to scramble or *Sp5* siRNAs (n=10). **Supplement to Figure 5E.**

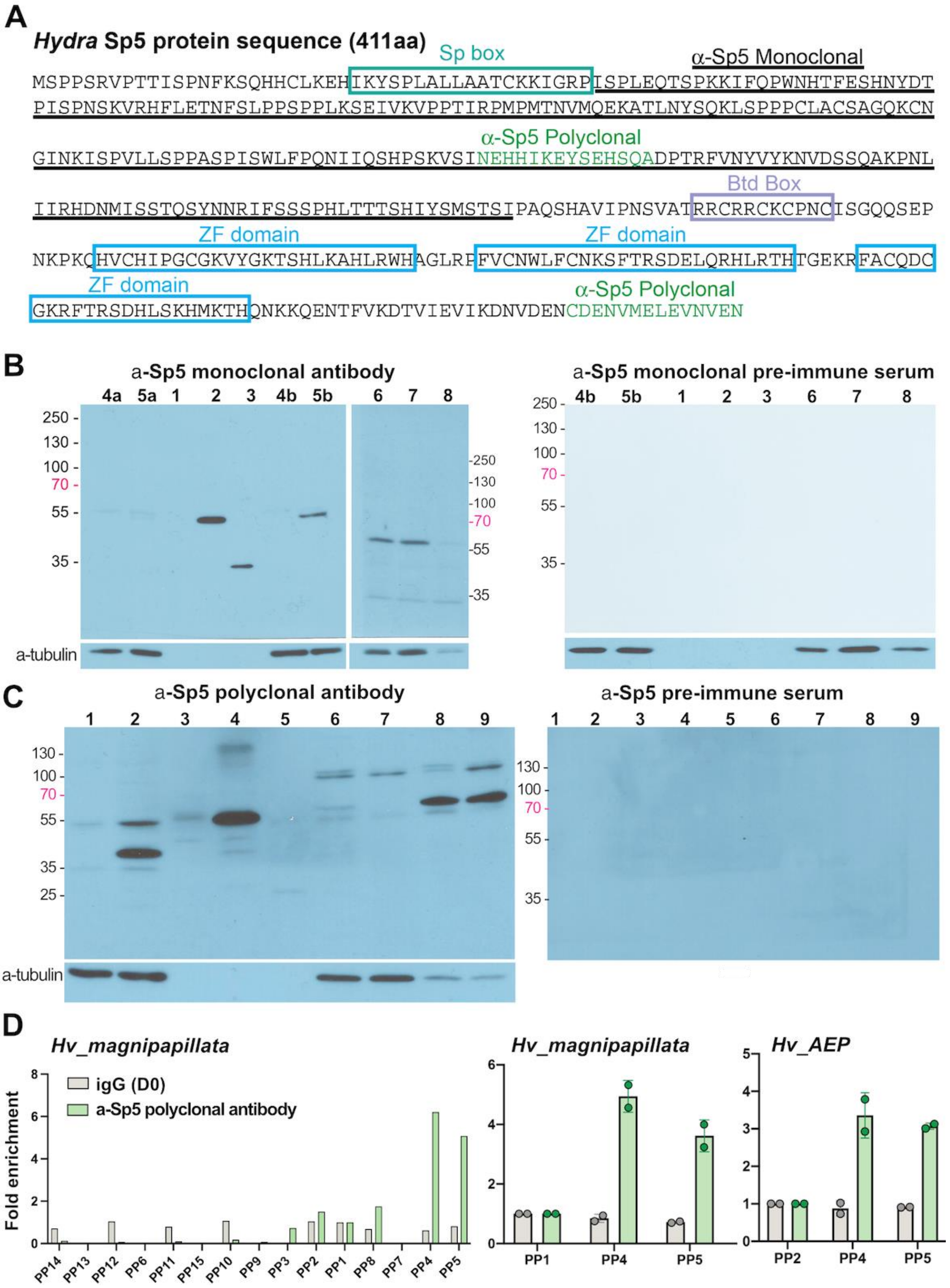

Figure S18. ChIP-qPCR analysis of the Sp5-binding sites in the *HySp5* promoter using anti *HySp5* antibodies.
(A) HySp5 protein sequence with the Sp box (green), Buttonhead (Btd) box (purple) and zinc finger (ZF) domains (blue). The 218 AA long sequence of HySp5-218 protein used to raise the monoclonal anti-Sp5 antibody is underlined, the peptides used to raise the polyclonal anti-Sp5 antibody are written green. **(B)** Western blot analysis testing the anti-Sp5 monoclonal antibody (left) and the pre-immune serum (right) against the Sp5 protein either TNT-produced (lane 2; empty control: lane 1), or synthesized as recombinant HySp5-218 protein (lane 3, 24.5 kDa), or expressed in HEK293T cells (lane 5b; empty control: lane 4b), or present in *Hv_AEP2* nuclear extracts (NEs) prepared from whole animals (lane 6), apical (lane 7) or basal (lane 8) halves. **(C)** Western blot analysis testing the anti-Sp5 polyclonal antibody and the pre-immune serum against the Sp5 protein either expressed in HEK293T cells (lane 2; empty control: lane 1), or TNT-produced (lane 4; empty control: lane 3), or synthesized as recombinant HySp5-218 protein (lane 5), or present in *Hv_AEP2* NEs prepared from whole animals (lane 6), apical region (lane 7), body column (lane 8) or basal regions (lane 9). In B and C, the loading was tested

by stripping the membranes and reprobing them with the anti-alpha tubulin antibody. **(D)** Enrichment in Sp5-binding along the *HySp5* promoter measured by ChIP-qPCR with *Hm-105* extracts using the anti-Sp5 polyclonal antibody and 15 primer pairs as depicted in **Figure 6C** (left), or restricted to the PP1, PP4 and PP5 regions (middle). ChIP-qPCR analysis of the PP2, PP4 and PP5 region when using *Hv_AEP* extracts and the anti-Sp5 polyclonal antibody (right). Note that in all conditions an enrichment is only detected in the PP4 and PP5 proximal regions. **Supplement to Figure 6.**

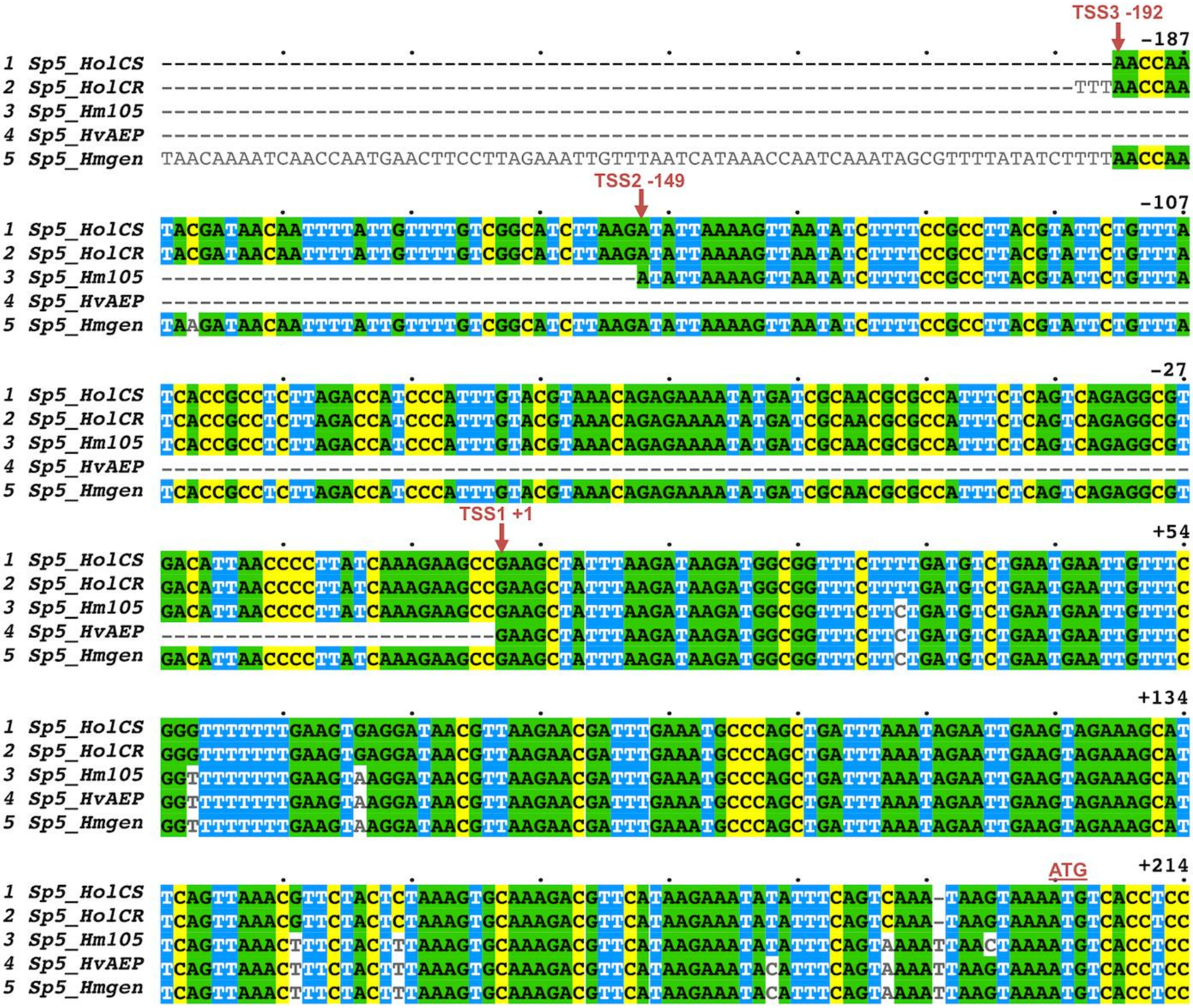

Figure S19. Mapping the putative *Sp5* Transcriptional Start Sites (TSS)

The *Sp5* cDNAs sequences were retrieved on HydrAtlas, https://hydratlas.unige.ch [37] for the Cold Sensitive or Cold Resistant *H. oligactis* (*HolCS:* S034511c0g1_i01*, HolCR*) and *H. vulgaris* (*Hv_AEP:* c16537_g1_i01) strains, on NCBI for *H. magnipapillata (Hm105:* XM_004206770.3*)*, then aligned to the *Sp5 H. magnipapillata* genomic sequence (Sp5_Hmgen) with Muscle Align (https://www.ebi.ac.uk/jdispatcher/msa/muscle) selecting a ClustalW output format and finally visualized with MView 1.63 (https://www.ebi.ac.uk/jdispatcher/msa/mview) [38]. Putative transcriptional start sites (TSS, indicated with vertical red arrows), were deduced from the 5' end of cDNAs from *Hv_AEP* (TSS1, position +1), *Hm105* (TSS2, position -149) and *H. oligactis* (TSS3, position -192). **Supplement to Figure 6E.**

(A) *Wnt3* upstream sequences from *Hm-105* Hydra

```
-2149    gagttgaagagcaagaaaacgaatgattgtaaattgtacatgtcgattgtgaaaattgactataaataattattaaaaatgtagattttatgtgcgagga
-2049    aaacatgaagaccatagaaaactccaaagcactgatgttcactgaaaaaactagataaataaaacgattttaactaagaggaacagtaaaaagatgcaac
-1949    ttagctaaatgatgagtaacgcgaaatagagttttggttgatggaaaaaaatatcgatcgctaacttgaacagcgaacatgtagtttttgttgctactgga
-1849    taccaaaccatggtaataatgctatcctttttttaattttaatttgactattttttaaatcttaagcccgcgctctccatcggtaaaaaagaagaca
-1749    atggcaaccctaaattaatgtatgtatgtaaaaggtcatcatatattaacatattataataaaatattatttatgttttaatactatttcttatatttt
-1649    tgattttcataaaaaatgatttgtttttatagactactattattatcatattatttttatatttcatttttatgtttttattactaatataactattat
-1549    taatctaaaacagtggaataaacatttagaatattgttgacttcatattcgcctaaaaagcatatgtttacatttttattaagtaattatctttgtttg
-1449    gcatttaataatattaaaaagtattcgtcaccaaaactctgcttttcaaatttataaatttatgttaacgttaaaacaatgcatcaatgcattgaacgt
-1349    tttttaatggaaaaattaacaattttgctatctttataagtttgtttagtttcaagttttatataatgtagcttgagtgtaaaaatattatttgagtct
-1249    aacaataaatctaaaataactttttgttgatgctagattttatacatctttaacagctatggcgccaaaagagacagcgggtgttttattgagctacgca
-1149    gttattgtttttttaaattcagtctttatcaaatgttaacaaataaaaatcctgattgcatcccaatcagctcatttggaatatgttatgaccaaccaaa
-1049    ttgttggcaaagtagttgtaaattatgatgtcatcttcttataggaaaatgacatcagtaaataatcgtcagaaaaaaacgtcagaaaacaagtgacgta
- 949    ttataattttgtttataatatacatatatatatataaaactcatcgatgacaaaacaatgaccaataaaatattcctttgatagagtctcctaatatgtt
- 849    atcttggtaatttgtacaattattttattaaatgtttatcaattaatgataaatttaaacttatagagatatttgcaatgttgataaaaaagatgtcgat
- 749    aacatattaacgaaatagtatatgaatataaacatataatattatcatatatattatgcacatcaagttttatgtttactgacgtttccctgccttctaa
- 649    ctcctacctgcacaaaaagcttgaactttatattttttaattagtataagcaaatagacgtcttaaagttaaatcgatacttctttaaaatctttacaa
- 549    acattaccatgtgaaatatttggcttaatatatcagaacagctatatcaatttcactgtaattttatcgcactgtgcttattattagatattcaataacc
- 449    cattgtgaaaaggcttaaacccgttttactttgttgctttaaaggtttgatttaactaaccgctaatcattataaaaagttataaatttataaaaattt
- 349    atctctgcaaaaaaagatttaacattaacacaactactacaaaaatctatctcaggatactgtcaaaaagttaaacaggtcattaaagttaactttgtc
- 249    aaagcggtatccctattccaaaagtttcaacgtaaccgtgtcaattctttacatagcaaatagaaagggtcaccgattaaagaaaataataaaaggattc
- 149    acacgtgctaatcgtgcgctttaaagatgactgattgtttctaactttatttgaaatttcacaagaaaagttttcacattaattttaaacacaatgcaa
-  49    aaataaacgcaaagaatatgcagttttaaaacatgcatttttaaagaaa
```

(B) *Zic4* upstream sequences from *Hm-105* animals and *Zic4* coding sequences from *Hv_Jussy*, *Hv_AEP*, *H. oligactis (CS)*

```
-3270    ccatgtcggttgtgtcttatagcgacttcgactttttatttctccaaaaaaacttccgacataaactagactttgagtaaagtcgaagagttgtttat
-3170    ttatattcttctttctgttttatttgctggtaaagtttttcaataaaaattatcaactgaggattaactgcaataaaaaagtactatctgtaaataaaa
-3070    taataataataataaataaaaaagatggtaaaaatttacttaagtaataataaaaaactttttgttcattaaaatatatatatatagttgttttgtaat
-2970    aatttttaaatttttacaatacatatcccttatctctgttaagccaaatgttttccgttacgaaatccttatatattgtgttatttttaaagtttacg
-2870    cgacaggaagctagtaatttaatatctttaagtttttagattttcaagtcaatatttctcttttactaataattttatgaaaataatttgttgtttttg
-2770    ttttgtcgttgagattggtacaataaaacatgaaaaaggcaaatttccgattaacaaagctttggattttgataaagaaaaaaaaaatccaaatttatta
-2670    tttcacatgaaaggtgcgaaagattagcggagttttgttttgtttttgaaataatacaattacaaattctttttagaaaatgcggattagaatttattaa
-2570    aatgacattaacactgttttgagtctaagaaacgtaaaatatgctagttatttttacaacaagcaggtaaaacaataaaaatggcctttaaagtaatttt
-2470    gagtttatttcattgttgttagctcaatcgatactagttcagtaaaatacgtttatttaaagtattttactaaaattatgctctttaatattcgaaaaaa
-2370    gaaaacaaactttttccaaaaatctaacatttttcattactcaaaaaaaaactttctttttgcgctttacgcagcgctacaaaaaatatgtcgctaagta
-2270    ctaaaattaaaaaaaacaaaaaaacaacgtaattacataacatgacgtaatgcaactttaatacgcatttgcagtgaatactttattatttatatatata
-2170    tatatatactcacacatacgtatacatatatatgtataatcaaatacgtaaatagatatagatataatatatgaatatattataagtttaatttattcta
-2070    ttgttattgatttttaaaagaaagtcagaaaatcttaaactgaaagcaaaaaaaaaactttttcaataatatactttatcattattttgttgttgttgt
-1970    tatgattgttattataattataataataactttattattgttattattattattgtaatcgtttatgaaaacttgatggttgctaaagatttaagg
-1870    taaataaatcgcccctctcgctattgatttgtttatttaaataacgggcattggcgtgaaattaatggcagcttatcatacgaattgctatacattca
-1770    aagaattttgttagtacccatgagtttattttgaggtgacccatgctgttctcattaatctaaaaccattggtttttgattaaaaggatcatcgctca
-1670    aataaagtgttttactcaggcgtgtgataactgtattactaagacttcctcgatataaacttgcatcaaattatttctgaataatgattattgtcaaaaa
-1570    caaagacacagttgactaaattcctaacttactttgtattattttttgtctgtaattttatagaccgatatgttacttttgctacatgtgttagaagttg
-1470    tttgagaattcaaactgagtgaaaaaaatggacaaataaaacaaaataaaacgcgtatgtcctgctacctttttatagctgttttaaataaagtttcttt
-1370    tggtattctaatgttgtgacttctaaaaaaattatgcaatcaaagattatcgacgcaacgataagtcaattaaaggaaaggggtgctacatatgagacaa
-1270    cacatgtgtgttttacattttaaccggtttaacgtcactccgaacaaagtttaaattaatgcgaacgttttattttgtagctaaacatctgagttaaa
-1170    aatgcttgcgcacgttcttttaacaaaaacagctcatcttattgtttttactttttattttttattaaattaaaaaaaatataattaaaaaatatttttgt
-1070    aagaatcttaacagatattttacgataaaatttcttatctaaagtatcgttttcaggttactcgcgtagacttataatttcaataaagattacccaatt
- 970    tgtctaattagcataacaattaattataagtaaataaccggagataataagtaaataacctgagataaaaacctcaaagagtacagcagattggaaatca
- 870    ttttttttttttcaagaaaccgcgcgcattcactcgtataccttaattgtataatcttttagttaaacaagcaccaaaaccggaacaaaggtagtagta
- 770    tacaatcaaagcaagctttgatatctctaatatttttttcaattttttttttatccacttatttgtctgagctatttctttaccttaattacgctataaa
- 670    atttcttctcgacaagcggcgtagctttgagtttatagctcttttctgaagtctaattccttctgtttttattacaatcctgactaagcttcctcaaag
- 570    tcaaggaaaacaaatgcagttaaaaagaaaataatccttgtaaacacgtttcatccttcataaaaaacgtacgctaataaagctttttttgtttacagtta
- 470    agcaagctaatttagaatttcagaattactctttgcaaaaaaaaaaaaaaaaaaaaaaaaaaaaaaaaaaaaaaaaagtaataataataattagcc
- 370    acgtcattttgcaaccagattaaaatccaggaaatctcaatgaaattatttgggaggaagttgtatatttctaaaatttagcagaacttatattagcgta
- 270    tatcttcgacacacataaacttactgtgatattacaaaatgactcaattgagttagaaataaaatcaaataaataaactaaatttcccaatactacaaa
- 170    gcaggatctgaaaggttcttttaaggattctcgcttcatactttttttgttttgtattatttttgctcccgttaattaggaaataaactaccatgcagatt
-  70    aattatgggtataattgaaagttcgtgcagttttattcttcgtcaaattaaatcaataatttagttctat
```

TSS1 *(Hv_Jussy)*
\+   1 ttcgttttattactataaatcctctaaagaacattttttgcttgttttctttttaaagatctcattttaattttttaatacccccgctatgatgtgattggat
Hv_Jus **TT**CGTTTTATTACTATAAATCCTCTAAAGAACATTTTTGCTTGTTTTCTTTTTAAAGATCTCATTTTAATTTTTAATACCCCGCTATGATGTGATTGGAT

TSS2 *(Hol_CS)*
\+ 100 gggatcaaatgtgattggttaaaaaagtttgaataagtatcaagaactgtcaatcaaaataaatgagcggccaatcaggttagttataaatccttattag
Hv_Jus GGGATCAAATGTGATTGGTTAAAAAAGTTTGAATAAGTATCAAGAACTGTCAATCAAAATAAATGAGCGGCCAATCAGGTTAGTTATAAATCCTTATTAG
Hol_CS ---**GT**C**G**AATGTGATTGGTTAAAAAAGTTTGAATAAGTATCAAGAACTGTCAATCAAAATAAATGAGCGGCCAATCAGGTTAGTTATAAATCCTTATTAG

TSS3 *(Hol_CR, Hv_AEP1)*
\+ 200 caacataatccctacacatagacagatgtaccatacataatggaaaggtaaaattgatttgtgcccaaaagcatttaaaagtctcaatttacatccgaat
Hv_Jus CAACATAATCCCTACACATAGACAGATGTACCATACATAATGGAAAGGTAAAATTGATTTGTGCCCAAAAGCATTTAAAAGTCTCAATTTACATCCGAAT
Hv_AEP -----------------------------------**CA**TAATGGAAAGGTAAAATTGATTTGTGCCCAAAAGCATTTAAAAGTCTCAATTTACATCCGAAT
Hol_CS CAACATAATCCCTACACATAGACAGATGTACCATACATAATGGAAAGGTAAAATTGATTTGTGCCCAAAAGCATTTAAAAGTCTCAATTTACATCCGAAT

\+ 300 gacaccgtatttcagtcacagtcagttggaagcagcgttctagacgtatcagagggttatctagttgataattgaatgtgacattgttttattaaatata
Hv_Jus GACACCGTATTTCAGTCACAGTCAGTTGGAAGCAGCGTTCTAGACGTATCAGAGGGTTATCTAGTTGATAATTGAATGTGACATTGTTTTATTAAATATA
Hv_AEP GACACCGTATTTCAGTCACAGTCAGTTGGAAGCAGCGTTCTAGACGTATCAGAGGGTTATCTAGTTGATAATTGAATGTGACATTGTTTTATTAAATATA
Hol_CS GACACCGTATTTCAGTCACAGTCAGTT**A**GAAGCAGCGTTCTAGACGTATCAGAGGGTTATCTAGTTGATAATT**A**AATGT**A**ACATTGT**G**TTATTAAATATA

\+ 400 aaagtatattttttatgcagcgtttgtattgtggtgtt-------acctcattaaatatctacaaattgttcctatattaaaacctaattatttatctaga
Hv_Jus AAAGTATATTTTTATGCAGCGTTTGTATTGTGGTGTT-------ACCTCATTAAATATCTACAAATTGTTCCTATATTAAAACCTAATTATTTATCTAGA
Hv_AEP AAAGTATATTTTTATGCAGCGTTTGTATTGTCGTGTT-------ACCTCATTAA**G**TATCTACAAATTGTTCCTATATTAAAACCTAATTATTTATCTAGA
Hol_CS A**C**A**T**TAT**T**TT**C**TTATGCA**A**C**A**TTTGTAT**A**GTGGTGTTA**AA**TCATACCTC**G**TTAAATATCTACAAATT**A**TTCCT**T**TATTAAAACCTA**G**TT**G**TTTAT**TGG**GA

\+ 500 aacttatttattaaatatctgtttcatta-aa**atg**gagcatcgtttacaaagttcaacattaggacattatccaaacgttccacggtactattacttaga
Hv_Jus AACTTATTTATTAAATATCTGTTTCATTA-AAATGGAGCATCGTTTACAAAGTTCAACATTAGGACATTATCCAAACGTTCCACGGTACTATTACTTAGA
Hv_AEP AACTTATTTATTAAATATCTGTTTCATTA-AAATGGAGCATCGTTTACAAAGTTCAACATTAGGACATTATCCAAACGTTCCACGGTACTATTACTTAGA
Hol_CS A**G**CTTATTTATT**T**AATATCTGTT**A**CATT**GA**A**C**ATGGAGCATCGCTTGCAAAGTTCAACATTAGGACATTATCCAAACGTTCC**G**CG**A**TATTATTACTTAGA
M E H R L Q S S T L G H Y P N V P R Y Y Y L D

Figure S20. Putative *Zic4*-binding sites in the *Hydra Wnt3* and *Zic4* genomic sequences

(A) Putative Zic-binding sites in the 2’142 bp *Wnt3* genomic sequence from *Hm-105* animals at positions -1781 and -1177 (highlighted in blue). **(B)** Putative Zic-binding sites in the 3’420 bp *Zic4* upstream genomic sequences (written lowercase) from *Hm-105* animals at positions – 1863, - 1824, -1744, -1289, -107 and +77 (highlighted in blue). This sequence is available in the Zic4-2505:GFP reporter construct positions 5’200 up to 8’993 (www.addgene.org/193001) [34]. *Zic4* cDNA sequences from two *H. vulgaris* strains (*Hv_Jussy, Hv_AEP1*) or *H. oligactis* (CS strain) are written uppercase. See accession numbers in **Table S1**.
